# Exploring A‐Site Chemistry of A_2_TlInF_6_ (A = Na, K) Double Perovskites: Structural, Optoelectronic, Elastic, and Thermoelectric Properties

**DOI:** 10.1002/open.70342

**Published:** 2026-11-11

**Authors:** Wafa S. Aljuaid, Imran Shakir, Shah Zeb Ullah, Shams u Zaman, Johar Zeb, Syed Hatim Shah, Muhammad Arif, Dereje Gelanu Dadi

**Affiliations:** ^1^ Department of Chemistry Taif University Taif Saudi Arabia; ^2^ Department of Physics Faculty of Science Islamic University of Madinah Madinah Saudi Arabia; ^3^ Department of physics Government postgraduate College Kohat Kohat KP Pakistan; ^4^ Department of Physics Kohat University of Science and Technology Kohat Pakistan; ^5^ Department of Physics School of Physics and Astronomy Yunnan University Kunming China; ^6^ School of Physics and Electronic Information Yunnan Normal University Kunming Yunnan China; ^7^ Department of Physics College of Natural and Computational Science Dambi Bollo University Dambi Dollo Ethiopia

**Keywords:** AIMD simulations, density functional theory, double perovskites, electronic structure, optical properties, thermodynamic properties, thermoelectric properties

## Abstract

First‐principles density functional theory (DFT) calculations were performed to investigate the structural, mechanical, electronic, optical, thermodynamic, and thermoelectric properties of double perovskites A_2_TlInF_6_ (A = Na, K). The tolerance factor, formation energies, Born–Huang criteria, and Gibbs free energy confirm the structural stability and energetic favorability of the cubic phases. Ab initio molecular dynamics simulations at 300, 500, and 700 K further demonstrate their structural integrity and finite‐temperature stability. Both compounds exhibit ductile behavior and elastic anisotropy. Without spin–orbit coupling (SOC), the indirect bandgaps are 2.41 eV for Na_2_TlInF_6_ and 2.68 eV for K_2_TlInF_6_. Including SOC, the PBE‐GGA/TB‐mBJ bandgaps are 2.37/5.05 and 2.64/4.75 eV, respectively. Optical spectra across 0–13.5 eV reveal strong ultraviolet‐region absorption, with electronic transitions primarily involving Tl‐*s* and F‐*p* states. Thermodynamic analysis shows decreasing Gibbs free energy, increasing entropy, thermal expansion, and decreasing Debye temperature with increasing temperature, reflecting enhanced thermal vibrations. BoltzTraP calculations predict maximum thermoelectric figures of merit (ZT) of 0.65 for Na_2_TlInF_6_ and 0.58 for K_2_TlInF_6_ at 800 K. Overall, these findings highlight their potential for ultraviolet optoelectronics and high‐temperature thermoelectric energy conversion and provide a basis for further theoretical and experimental investigation.

## Graphical abstract

First‐principles DFT calculations reveal the structural, mechanical, electronic, optical, thermodynamic, and thermoelectric properties of A_2_TlInF_6_ (A = Na, K) double perovskites. Both compounds exhibit favorable stability, ultraviolet optical response, and promising high‐temperature thermoelectric performance, with maximum (ZT) values of 0.65 for Na_2_TlInF_6_ and 0.58 for K_2_TlInF_6_ at 800 K, highlighting their potential for optoelectronic and energy‐conversion applications.

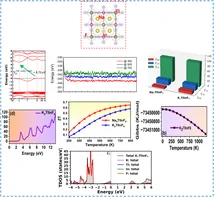

## 1 Introduction

The increasing global demand for renewable energy has driven research toward materials that are both viable and efficient for recent energy applications [1,2]. To address these challenges, renewable sources such as biomass and photosynthesis‐based systems are actively being explored for energy conversion applications [3]. In parallel, nontraditional catalytic approaches, including electro‐, photo‐, plasma‐, and sonocatalysis, have gained significant attention [4, 5, 6, 7, 8, 9]. Recent studies in materials science continue to enable the discovery of compounds with improved physicochemical properties, while computational methods provide low‐cost and effective tools for predicting and designing novel materials [10, 11, 12]. Despite these developments, one of the major scientific challenges that remains is the identification of efficient compounds for energy conversion and storage. The inherent structural flexibility of perovskite‐type hydride materials opens the door to chemical substitutions and doping strategies. Such modifications can be used to tailor electronic properties for energy storage, enhance ionic mobility, and fine‐tune hydrogen release behavior [13, 14].

Another interesting class of hybrid organic–inorganic perovskites is emerging materials with excellent optoelectronic properties and high power conversion efficiencies in solar cells [15, 16]. Their tunable bandgap, long high charge‐carrier mobility, and carrier diffusion length make them promising candidates for next‐generation photovoltaic devices [17, 18, 19]. However, their practical application is limited by poor structural stability and lead toxicity [20, 21]. Therefore, significant efforts are directed toward developing lead‐free and environmentally benign alternatives [22]. The wide‐bandgap perovskites are actively sought for ultraviolet (UV) and visible optoelectronics with improved environmental compatibility [23]. In this framework, double perovskites A_2_B′B″X_6_ have emerged as promising candidates due to their robust crystal structure and nontoxic nature [24, 25]. Moreover, their compositional flexibility enables tuning of structural and electronic properties for advanced optoelectronic applications [26, 27]. Notably, alkali‐metal‐based halide double perovskites, where A is a monovalent cation (Li, Na, K, Rb, Cs), B is a trivalent metal (Bi, Sb, Y, In, Sc, Tl, Ga), and X is a halide ion (Cl, Br, I), exhibit excellent optical and charge‐transport properties suitable for photovoltaic and energy‐related applications [28, 29, 30, 31]. In recent years, lead‐free double perovskites have attracted significant attention due to their structural versatility, environmental compatibility, and nontoxicity. Among them, Cs_2_AgBiX_6_ (X = Cl, Br) has been widely studied since 2016 owing to its suitable bandgaps and potential in photocatalytic CO_2_ reduction and hydrogen evolution [32, 33]. Similarly, Cs_2_AgInCl_6_ has been explored for pollutant degradation and UV photodetectors, while Cs_2_AgSbCl_6_ shows notable photoluminescence efficiency and air stability [32, 34, 35], highlighting the promise of Cs‐Ag‐based perovskites for optoelectronic applications [36]. High‐throughput studies have also predicted Cs_2_InBiCl_6_ and Cs_2_InSbCl_6_ as lead‐free alternatives to MAPbI_3_ due to their favorable bandgaps [37, 38], although their synthesis is challenging due to In^+^ instability. In contrast, experimentally studied systems such as Cs_2_AgSbCl_6_ and Cs_2_AgInCl_2_ demonstrate promising optoelectronic performance [39, 40, 41], whereas Cs_2_CuBiX_6_ compounds are less suitable for photovoltaics due to indirect bandgaps [42]. Further computational studies suggest Cs_2_AgMCl_6_ (M = Al, Ga, In, Tl) as promising materials, with Ga‐ and In‐based compounds exhibiting ideal bandgaps (0.9–1.6 eV) superior to earlier Ag/Bi systems [43]. Among double perovskites, investigations into fluorine‐based double‐perovskite halides have attracted considerable attention owing to their promising thermal and structural stability, which distinguishes them from their organic–inorganic hybrid counterparts. Fluorine‐based double perovskites are gaining attention for their stability and tunable properties. Rb_2_XRhF_6_ (X = Li, Ag) compounds have been studied using the FP‐LAPW method with mBJ potential [44]. These findings show that strong UV absorption in both halides suggests use in lasers and UV‐protection devices, and their high conductivity and ZT values point to thermoelectric potential. Similarly, Saif M.H. Qaid et al. reported that Rb_2_TlRhF_6_ has emerged as a compelling candidate, as the incorporation of Rb^+^ cations has been shown to significantly enhance the overall performance of perovskite‐based devices while simultaneously improving their thermal and structural robustness. For instance, a first‐principles study of the rubidium‐based double‐perovskite halide Rb_2_TlRhF_6_, employing the TB‐mBJ potential, confirmed its structural stability through negative formation energy and a favorable tolerance factor and revealed a direct bandgap of 3.2 eV at the (L–L) symmetry points, thereby establishing its semiconducting nature [45]. It is worth mentioning that the electronic structures of the aluminum and thallium analogs are similar, making them attractive alternatives to In‐ and Ga‐based systems. Indium‐based fluoride double perovskites have a wide range of applications and have emerged as a promising class of materials in the fields of photovoltaics, optoelectronics, and catalysis. For instance, Cs_2_InBiF_6_ was investigated; the observed direct bandgap nature and optical characteristics revealed that the material exhibits high polarization in the UV region, which makes this material suitable for optoelectronic technologies [46].

Perovskites that incorporate Tl have been experimentally and theoretically shown to exhibit improved light absorption and carrier mobility, highlighting their potential for efficient energy conversion [47, 48, 49]. Simultaneously, potassium (K)‐ and sodium (Na)‐based perovskites have attracted interest for their ability to enhance structure stabilities; the B′‐site dominates the valence band, and the B′′‐site dominates the conduction band, while the X site plays a key role in tuning the electronic bandwidth and bandgap. Accordingly, such compounds have been reported in the literature, including K_2_AgXI_6_ (X = Sb, Bi), KXCl_3_ (X = Ca, Mg, Be), K_2_AgAsX_6_ (X = Cl, Br), KTaO_3_, KMnF_3_, K_2_PdX_6_ (X = Cl, Br, I), K_2_NaTlX_6_ (X = Cl, Br, I), and Na_2_AgGaY_6_ (Y = Cl, Br, I) [50, 51, 52, 53, 54, 55, 56, 57]. Despite recent developments, electron–phonon interactions and anharmonic effects in such compounds remain insufficiently explored. Metal halide perovskites show strong potential for photovoltaic, optoelectronics, and thermoelectric applications such as converting light into electricity (solar cells) and heat into electricity (thermocouples) for production and environmental remediation [58].

Despite extensive research on A‐site engineering in oxide and chloride/bromide double perovskites, the significance in Tl‐In remains unclear. Therefore, the study related to fluoride‐based double perovskites is mostly unexplored. We present a comprehensive first‐principles analysis of Na_2_TlInF_6_ and K_2_TlInF_6_ to examine the impact of Na^+^/K^+^ substitution on structural stability, extending the study to fluoride systems where F^−^ plays a key role in tuning structural, mechanical, electronic, optical, thermoelectric, and thermodynamic properties. The findings demonstrate the impact of electrostatic interaction with the size of the A‐site cation on bandgaps, elastic anisotropy, thermoelectric efficiency, and Debye temperature. These results enhance the foundational comprehension of Tl‐containing fluoride double perovskites, while their practical uses require careful evaluation of thallium toxicity and environmental hazards.

This work comprehensively investigates the characteristics of A_2_TlInF_6_ (A = Na, K) fluoroperovskites as efficient and sustainable semiconducting materials for next‐generation solar energy technologies by engineering their crystal skeleton and electronic properties. The cubic phase that crystallized in the *Fm*‐$\bar{3}$ *m* structure was investigated by utilizing first‐principles calculations with the generalized gradient approximation (PBE‐GGA). Their electronic, optical, and thermoelectric features were studied to explore the impact of the A‐site cation. The results reveal appropriate electronic and light–matter interaction characteristics, making the investigated fluoroperovskites compounds promising candidates for future applications in advanced energy conversion and optoelectronic devices.

## 2 Methodology

First‐principles calculations have been carried out within the computational package of WIEN2k based on density functional theory (DFT) [59]. The full‐potential linearized augmented plane wave (FP‐LAPW) approach was utilized to analyze the interaction between the valence electrons and atomic nuclei in a crystal lattice. This approach is an all‐electron first‐principles technique, which treats valence and core electrons explicitly without employing shape approximation for either the potential or the charge density. This makes it the most accurate method for evaluating the electronic a related property of solids. The FP‐LAPW method is the most reliable DFT method and has been utilized to build a comprehensive understanding of the intrinsic features of crystalline materials [60]. The generalized gradient approximation (GGA‐PBE) was employed to relax the structure and minimize the total energy [59]. A more realistic evaluation of the electronic bandgap was estimated using the GGA‍‐‍PBE. GGA‐PBE including spin–orbit coupling (SOC) and Tran–Blaha modified Beck–Johnson (TB‐mBJ) exchange‐correlation potential including SOC, which improve the bandgap estimation important for electronic property calculations [61]. This semilocal functional reduces bandgap errors compared to the conventional GGA formulation and provide bandgap values close to experimental data. The muffin‐tin radii (RMT) values were fixed to 2.45, 1.65, 2.20, and 1.67 Bohr radii (*a*_0_) for X (Na, K), Tl, In, and F, respectively. The plane‐wave (RMT x *K*_max_) cutoff parameter was set to 7.0 to get a possible converged basis set. The energy level of −7 Ry was used as a criterion to differentiate between core and valence states. The criteria were chosen as 10^−5^ Ry for total energy, 10^−4^ e for charge density, and 1.0 mRy/a.u for atomic forces. The self‐consistent field (SCF) iterations were carried out with high numerical accuracy, namely, an angular momentum expansion (*l*_max_) and a 1500‐k‐point mesh in the irreducible Brillouin zone. The crystal structures, atomic arrangements, and bond geometry were visualized using the VESTA software [62]. The quasi‐harmonic Debye model was employed within the Gibbs2 code to estimate the thermodynamic features of compounds. Utilizing energy–volume dataset derived from density functional theory calculations enables the evaluation of key thermodynamic properties such as bulk modulus, Debye temperature, thermal expansion behavior, and heat capacity, offering a comprehensive understanding of the materials’ mechanical robustness and thermal stability. Furthermore, the BoltzTraP code was utilized to evaluate the thermoelectric properties including Seebeck coefficient, electrical conductivity, and thermal conductivity, under the constant relaxation time approximation with a relaxation time of 10^−14^ s.

## 3 Result and Discussion

### 3.1 Structural Properties

The investigated materials crystallize in a double‐perovskite crystal architecture represented by A_2_B′B″X_6_, consisting of cationic sites and an anionic framework. This material exhibits a highly symmetric cubic structure belonging to the *Fm*‐$\bar{3}$ *m* (space group # 225) crystallographic phase [63]. The unit cell contains four different atomic species: A = (Na, K), Tl, In, and F, where [TlF_6_]^5−^ and [InF_6_]^3−^ octahedra are alternated and related to each other by common atoms of fluorine positioned at the corners of the octahedron. The centered alkali‐metal atoms (Na or K) are present at the A‐site positions in the crystal structure, which stabilize the three‐dimensional frame double perovskite structure. The atomic positions in the crystal lattice, based on Wyckoff positions, are presented as follows: K/Na 8c (0.25, 0.25, 0.75), Tl 4a (0, 0, 0), In 4b (0.5, 0, 0), and F 24e (0.22, 0, 0). Figure 1 (left side) gives the detailed crystal skeleton of A_2_TlInF_6_ (A = Na, K).

**FIGURE 1:**
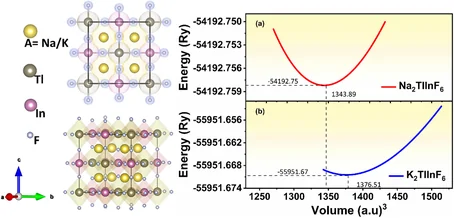
(Left side) prototype crystal structure, and (a,b) optimization plots for A_2_TlInF_6_ (A = Na, K).

The geometry of cations and anions plays a key role in determining the structural stability and electronic properties of perovskite materials. Therefore, a comprehensive evaluation of the structural parameters is required. The volume optimization method was employed to evaluate the total energy–volume relationship for the stable structural properties [64]. The computed dataset was fitted using the Birch–Murnaghan equation of state (Equation (1)) to determine the lattice constant (*a*_0_) of the equilibrium lattice structure, bulk modulus (*B*_0_), the pressure derivative of the bulk modulus (*B*′), and the ground‐state energy (*E*_0_). The calculated structural parameters are summarized in Table 1. The parabolic *E*–*V* curve displays the optimization steps of the unit cell, where the lower peak is the ground‐state energy versus volume of the unit cell, as presented in Figure 1a,b.

**TABLE 1:** Calculated lattice constant *a*_o_, bulk modulus *B* (GPa), equilibrium cell volume *V*_o_, ground state energy *E*_o_, tolerance factors, and formation energy of A_2_TlInF_6_ (A = Na, K).

Compounds	*a*_o_, Å	*B*, Pa	*V*_o_, (a.u)^3^	*E*_0_	$\tau_{1}$	*τ*_2_	Δ*H*_f_, eV/atom
Na_2_TlInF_6_	9.26	46.95	1343.89	−54 192.75	0.88	3.85	−2.81 eV
K_2_TlInF_6_	9.34	47.13	1376.51	−55 951.67	0.96	3.48	−2.90 eV



(1)

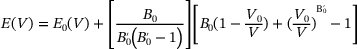




The calculated lattice constants expectedly increase from Na^+^ to K^+^, resulting in a slight increase in lattice dimensions. This trend is due to the difference in ionic radii between Na^+^ and K^+^ and is consistent with the overall behavior of perovskites, in which larger A‐site cationic substitution increases the lattice size. Although no experimental or theoretical data have been reported for these individual compounds, our results are consistent with reported studies of isostructural halide double perovskites, in which bismuth (Bi) is substituted by thallium (Tl). First‐principles evaluation of X_2_InBiF_6_ (X = Na, K, and Rb) reported in [58] follow the same trend with replacement of Na cation with K; the lattice constant increases from 9.72 to 9.78 Å, respectively. Similar dependence is also found in double perovskites Na_2_AgGaY_6_ (Y = Cl, Br, I) and K_2_AgGaZ_6_ (Z = Cl, Br) [65, 66]. It is evident that our findings are close to the previously reported trends, which have been used to validate them, supporting the consistency and reliability of the optimized structural parameters reported in this study.

The Goldschmidt tolerance factor (*τ*_1_) is an important empirical characteristic that was taken into consideration to assess the cubic structural stability of the investigated double‐perovskite compounds. This factor assesses whether a given combination of ionic radii can stabilize a perovskite lattice free from defects or structural distortions. For structural stability, the tolerance factor lies between 0.8 and 1.0 [67, 68]. To further evaluate the perovskite formability of the investigated compounds, the Bartel tolerance factor (*τ*_2_)) was computed in addition to the Goldschmidt tolerance factor. The Bartel criterion suggests that materials with (*τ*_2_ < 4.18) are anticipated to have a higher propensity for forming perovskite structures. The *τ*_1_ and *τ*_2_ for the examined compounds of A_2_TlInF_6_ (A = Na, K) are calculated as follows:

(2) $$\tau_{1}=\frac{r_{F}+r_{A}}{\sqrt{2}\left(r_{F}+\frac{r_{\mathrm{Tl}}+r_{\mathrm{In}}}{2}\right)}$$

(3) $$\tau_{2}=\frac{r_{F}}{\frac{r_{\mathrm{Tl}}+r_{\mathrm{In}}}{2}}-n_{A}\left(n_{X}-\frac{r_{A}}{\frac{r_{\mathrm{Tl}}+r_{\mathrm{In}}}{2}}/\ln\frac{r_{A}}{\frac{r_{\mathrm{Tl}}+r_{\mathrm{In}}}{2}}\right)$$

Furthermore, the chemical reactivity and energetic favorability of the compounds were evaluated by computing the formation energy (Δ*H*_f_). An exothermic reaction is indicated by a negative Δ*H*_f_, which suggests that the material is chemically less reactive and energetically favorable with respect to the elemental constituents, whereas a positive Δ*H*_f_ suggests instability [69]. The following relation [64] was used to compute the enthalpy of formation for the A_2_TlInF_6_ (A = Na, K) systems:

(4) $$\Delta H_{f}=E_{A_{2}{\mathrm{TlInF}}_{6}(A=\mathrm{Na},K)}\text{–}[2E_{A=\mathrm{Na},K}+E_{\text{Tl}}+E_{\text{In}}+6E_{F}]$$

In this expression, *E*_X2TlInF6_ is the optimized total ground‐state energy of the compound, while *E*_A_, *E*_Tl_, *E*_In_, and *E*_F_ are the total energies of the Na (or K), Tl, In, and F atoms in bulk structure, respectively. The negative formation energy as seen in Table 1 confirms the energetic favorability of the examined perovskites and explores their potential applications in thermoelectrics and optoelectronics.

Moreover, the influence of alkali‐metal substitution on the structural geometry of Na_2_TlInF_6_ and K_2_TlInF_6_ was investigated by comparing their lattice parameters, metal–fluorine bond lengths, and octahedral bond angles, as listed in Table 2. The lattice parameter increases from 9.26990 Å for Na_2_TlInF_6_ to 9.34430 Å for K_2_TlInF_6_, corresponding to an increase of approximately 0.80%. Similarly, the mean Tl─F bond length increases from 2.50287 to 2.52296 Å, while the mean In─F bond length increases from 2.13208 to 2.14919 Å. These changes indicate a modest expansion of the metal–fluorine coordination environments upon replacing Na with the larger K cation. Both structures exhibit reported F─M─F angles spanning 90°–180° and linear Tl─F─In bridging angles of 180°. The calculated bond‐direction deviations from the cubic reference axes are zero for both compounds. Therefore, the analyzed structures exhibit regular local octahedral coordination without detectable deviations from the selected cubic reference directions. These results suggest that alkali‐metal substitution primarily changes the lattice dimensions and metal–fluorine bond lengths, while the reported angular geometry remains unchanged.

**TABLE 2:** Comparison of lattice parameters, metal–fluorine bond lengths, octahedral bond angles, and bridging angles in Na_2_TlInF_6_ and K_2_TlInF_6_.

Structural parameter	**Na**_**2**_ **TlInF**_**6**_	**K**_**2**_ **TlInF**_**6**_
Lattice parameter = *a*, Å	9.26990	9.34430
Mean Tl─F bond length, Å	2.50287	2.52296
Mean In─F bond length, Å	2.13208	2.14919
Tl─F bond‐length range, Å	2.50287–2.50287	2.52296‍–‍2.52296
In─F bond‐length range, Å	2.13208–2.13208	2.14919‍–‍2.14919
TlF_6_: F─Tl─F angle range, °	90–180	90–180
InF_6_: F─In─F angle range, °	90–180	90–180
Mean Tl─F─In bridging angle, °	180	180
Mean Tl octahedral axis deviation, °	0	0
Mean In octahedral axis deviation, °	0	0

In addition, a material with dynamic stability is an important factor in both synthesis and practical applications, such as energy conversion and optoelectronic devices. It reflects its ability to resist external perturbations and lattice vibrations without changing its structure and phase. For ab initio molecular dynamics (AIMD) simulation, the VASP code was utilized using a 2 × 1 × 1 supercell. The dynamic and structural stability of the compounds Na_2_TlInF_6_ and K_2_TlInF_6_ is evaluated over a temperature range of 300, 500, and 700 K under the NVT approach, along with the evolution of temperature during the simulation as a function of time‐step in picoseconds, as shown in Figure 2a–d [70, 71]. The estimated result of total energy as a function of time‐steps indicates that both compounds maintain their equilibrium around a specific range and no sudden surge or drop is observed during the simulation up to 12 ps, as shown in Figure 2a,b. Similarly, time‐step‐dependent different temperature response reflects the stable range around 300, 500, and 700 K for both investigated compounds, as seen in Figure 2c,d. This signifies that the considered thermostat will be reliable and maintain the structural and dynamic stability of investigated compounds as illustrated in Figure 2a–d. Hence, AIMD simulation confirms the thermal and structure integrity over the investigated temperature range of 300, 500, and 700 K, validating that the studied materials are thermodynamically and structurally stable and can be synthesized experimentally. The stability of our investigated materials at 300 K is consistent with the results found by Aymane EL Haji et al. in [72]. Hence, our AIMD findings further support the finite‐temperature structural and dynamic stability of the A_2_TlInF_6_ (A = Na, K) compounds at 300, 500, and 700 K.

**FIGURE 2:**
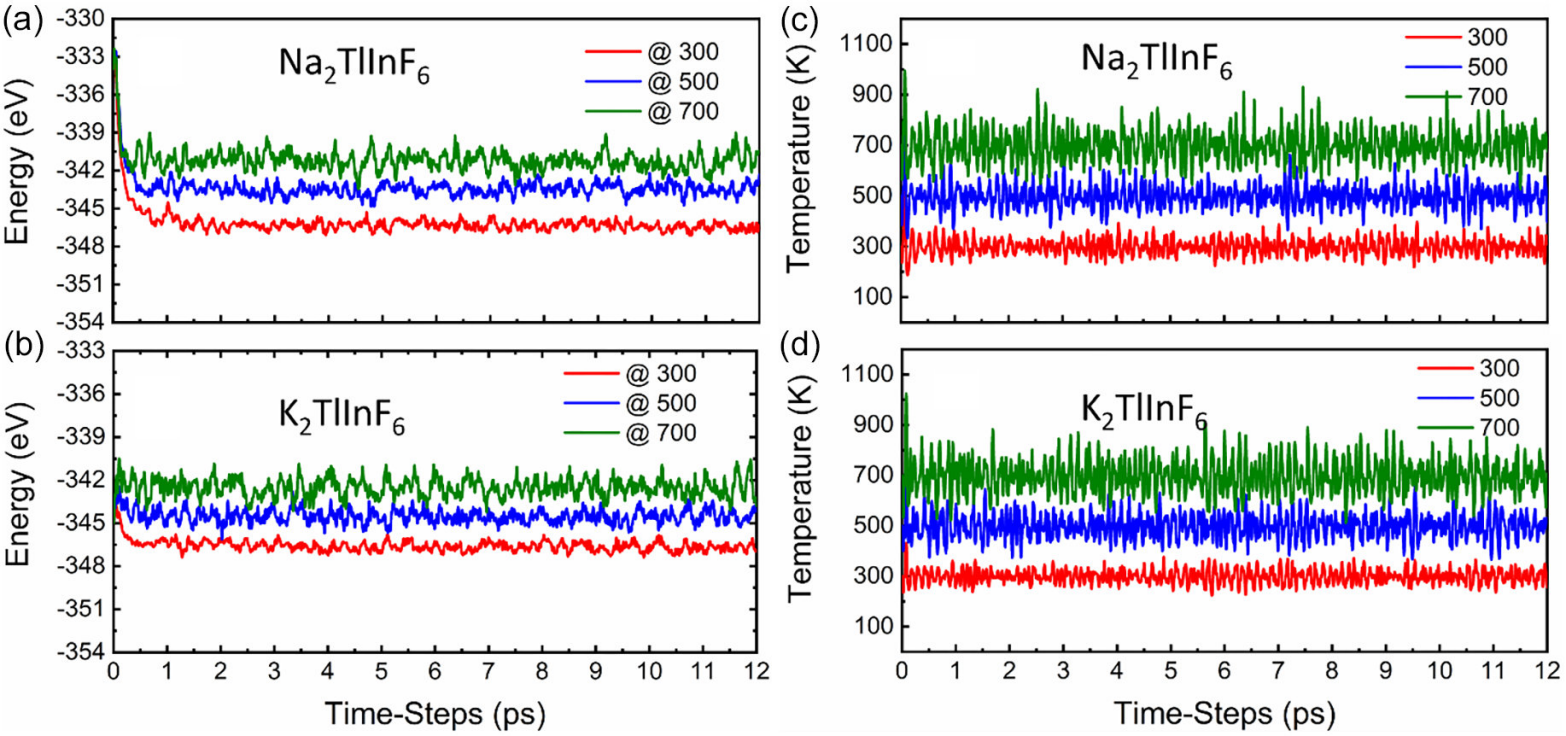
AIMD simulation results for A_2_TlInF_6_ (A = Na, K): (a,b) total energy as a function of simulation time at 300, 500, and 700 K; and (c,d) temperature observation during the simulations as a function of simulation time.

### 3.2 Elastic Properties

The elastic behavior of a material is useful for understanding its mechanical strength and structural integrity, and it finds applications in scientific studies as well as in practical use. Overall, elastic properties refer to a material’s capacity to withstand deformation under external forces, and the deformation is reversible when the stress is removed. This is a linear relationship between strain and stress, also known as Hooke’s law, and holds only within the elastic limit, where distortions are minimal. Once the deformation is beyond this level, the material experiences plastic deformation, resulting in irreversible alterations in its shape and size [73]. In this study, the elastic datasets of the A_2_TlInF_6_ (A = Na, K) compounds were calculated employing the Charpin finite strain method implemented in the WIEN2k code. The technique completely relaxes the crystal structures to reach equilibrium at nearly zero external pressure. This calculates the second‐order elastic constants by using the cubic elastic package [74]. The calculated three independent elastic constants (*C*_11_, *C*_12_, and *C*_44_) show the mechanical response of the cubic materials. In this case, *C*_11_ is the longitudinal compression resistance, and it is related to the hardness of the material; *C*_12_ is the interrelationship between the axial and lateral strain, and it links to the Poisson effect; and *C*_44_ is the shear resistance of the material (Figure 3). It is noted that all the values in Equation (5) are positive, as listed in Table 2, which proves that the studied compounds meet the Born–Huang criteria of mechanical stability of cubic systems [75]. This implies that Na_2_TlInF_6_ and K_2_TlInF_6_ are mechanically stable and retain their structural integrity under stress.

**FIGURE 3:**
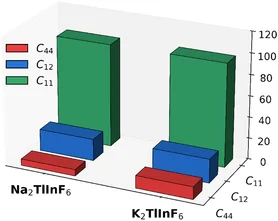
Elastic constants *C* *_ij_* of A_2_TlInF_6_ (A = Na, K).

(5) $$C_{11},C_{44}>0,\;C_{12}<B<C_{11},\;C_{11}-C_{12}>0,C_{11}+2C_{12}>0\;\;\;\;$$

The aforementioned calculation is used to determine the anisotropy and elastic moduli (*B*, *G*, *Y*, and *υ*) of the compounds listed in Table 3 using the relations given below [76]. The following Equations (6) – (12) are utilized to evaluate the important properties of the investigated materials.

**TABLE 3:** Elastic constants and related moduli for A_2_TlInF_6_ (A = Na, K).

Compound	*C*_11_	*C*_12_	*C*_44_	*B*	*E*	ν	*A*	*G*	*B*/*G*	*C*_12_–*C*_44_	*G*_v_	*G*_R_	Stability
Na_2_TlInF_6_	97.23	20.35	6.01	45.9	38.1	0.36	0.15	14.01	3.28	14.34	18.97	9.05	Stable
K_2_TlInF_6_	97.6	22.38	11.74	47.4	50.6	0.31	0.24	19.14	2.47	10.64	22.09	16.2	Stable

(6) $$B=\frac{C_{11}+2C_{12}}{3}$$

(7) $$E=\frac{9BG}{3B+G}$$

(8) $$\nu =\frac{3B-2G}{2\left(2B+G\right)}$$

(9) $$A=\frac{2C_{44}}{C_{11}-C_{12}}$$

(10) $$G=\frac{G_{v}+G_{R}}{2}$$

(11) $$G_{v}=\frac{C_{11}-C_{12}+3C_{44}}{5}$$

(12) $$G_{R}=\frac{5C_{44}\left(C_{11}-C_{12}\right)}{4C_{44}+3\left(C_{11}-C_{12}\right)}$$

Bulk modulus (*B*) is a measure of how a substance resists uniform compression and resists an increase in volume due to the presence of pressure. The evaluated metrics for Na_2_TlInF_6_ and K_2_TlInF_6_ are in good agreement with those projected by the Murnaghan equation of state, thereby demonstrating the validity of the structural optimization. The *B* metrics for Na_2_TlInF_6_ are slightly lower, which suggests that it is more compressible and flexible than K_2_TlInF_6_. The modulus of elasticity (*E*) and shear modulus (*G*), which are measures of stiffness and resistance to change of shape, respectively. The replacement of Na with K reflects that K_2_TlInF_6_ is mechanically stiffer than Na_2_TlInF_6_. The *B*/*G* ratio shows the ductile nature of the materials, as the values exceed the limit (1.75) for both compounds (3.28 for Na_2_TlInF_6_ and 2.47 for K_2_TlInF_6_), as seen in Figure 4a [77]. The Poisson’s ratio (*ν*) provides additional insight into the ductile/brittle nature of materials. The critical value is *ν* = 0.26, below which materials exhibit brittle behavior, and above which they are ductile. The calculated Poisson’s ratios for Na_2_TlInF_6_ (*ν* = 0.36) and K_2_TlInF_6_ (*ν* = 0.31) both exceed this threshold, confirming their ductile nature, which agrees with the *B*/*G* analysis [39]. Moreover, the Cauchy pressure (*C*_12_–*C*_44_) for both materials is positive, supporting their ductile character, as seen in Figure 4b. Directional dependence of the elastic properties is the Zener anisotropy factor (*A*), which has a value less than one, suggesting that the two compounds are elastically anisotropic, and their mechanical properties differ slightly in different crystallographic directions [40].

**FIGURE 4:**
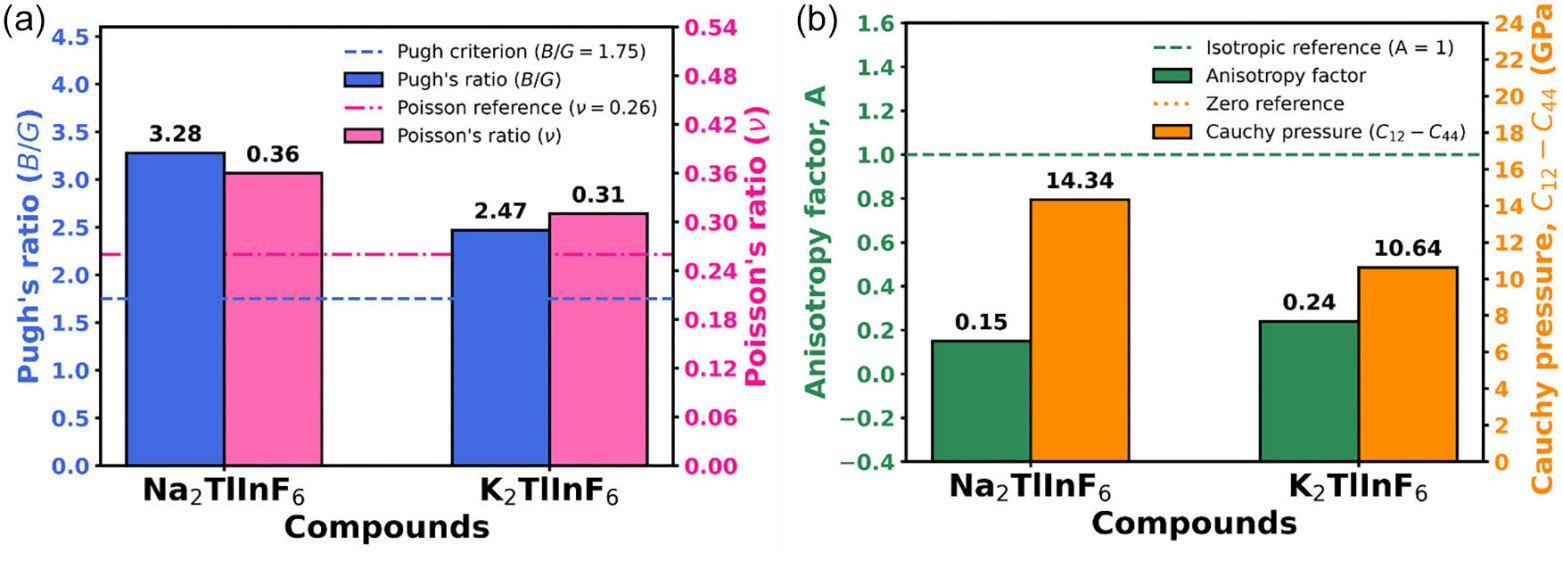
The calculated (a) Poisson’s ratio and Pugh’s ratio. (b) Anisotropy factor and Cauchy pressure for A_2_TlInF_6_ (A = Na, K).

### 3.3 Electronic Properties

The electronic structure of a material is very important in determining whether it is suitable for use in several technological applications. The energy band structures and the density of states (DOS) were calculated to develop a detailed analysis of the electronic behavior of the compounds. These studies aid in classifying materials as semiconductors, insulators, or metals based on their electronic transitions and bandgaps. All computations were performed using DFT with the PBE‐GGA, PBE‐GGA‐SOC, and TB‐mBJ‐SOC exchange‐correlation functional, which is much more accurate at estimating the bandgap than standard functionals including SOC due to the heavier atoms of In and Tl. In solids, electrons can occupy certain energy levels known as allowed energy states or energy bands. The region between allowed energy levels of valence and conduction bands, where electrons cannot exist, is called energy bandgap. Since all the simulations were conducted at 0 K, the Fermi level aligns with the maxima of the valence band. The band structure evaluations without SOC demonstrate indirect bandgaps of 2.41 and 2.68  eV for Na_2_TlInF_6_ and K_2_TlInF_6_, respectively. When SOC is included, the corresponding bandgaps are 2.37/5.05 and 2.64/4.75 eV and band structures remain indirect as calculated with PBE‐GGA, whereas the TB‐mBJ‐calculated bandgaps are direct in nature, respectively, as seen in Figures 5b,d and S1 and listed in Table 4. The electronic band structures show that the highest occupied valence states and the lowest unoccupied conduction states are located at different high‐symmetry *k*‐points, X and L, respectively. This spatial separation of the band extrema indicates that both materials have an indirect semiconducting bandgap. The X–L transition observed emphasizes the indirect electronic transitions that underlie their semiconducting nature. Additionally, we can observe that the bandgap systematically increases with increasing alkali metal atomic size (Na < K), i.e., between Na_2_TlInF_6_ and K_2_TlInF_6_, which agrees with the previous results on analogous halide perovskites [58]. The optical absorption enhances with increase in the bandgap and directly improves its electronic and optoelectronic functionality, making the investigated compounds promising for photonic devices and energy conversion applications.

**FIGURE 5:**
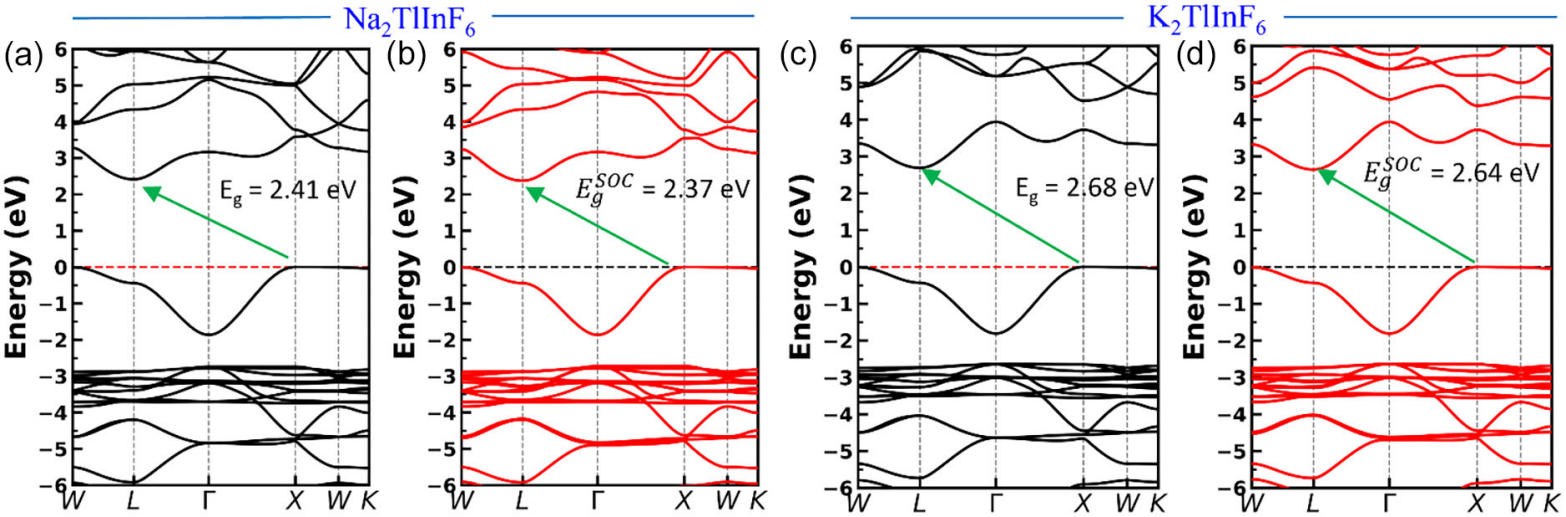
Electronic band structures of Na_2_TlInF_6_ and K_2_TlInF_6_ calculated using (a,c) PBE‐GGA and (b,d) PBE‐GGA with spin–orbit coupling (SOC), respectively.

**TABLE 4:** Estimated effective masses and bandgap by utilizing different functionals.

Materials	Effective masses						Bandgap		
	PBE‐GGA		PBE‐GGA‐SOC		TB‐mBJ‐SOC				
	*m* ^*^_e_	*m* ^*^_h_	*m* ^*^_e_	*m* ^*^_h_	*m* ^*^_e_	*m* ^*^_h_	PBE‐GGA	PBE‐GGA‐SOC	TB‐mBJ‐SOC
**Na**_**2**_ **TlInF**_**6**_	0.058	0.238	0.058	0.238	0.076	0.196	2.41	2.37	5.05
**K**_**2**_ **TlInF**_**6**_	0.057	0.094	0.057	0.094	0.081	0.274	2.68	2.64	4.75

Additional analysis of the electronic transitions between the VBM and the CBM through the density of states (DOS) was obtained, as illustrated in Figure 6a–f. DOS plots give a rich understanding of the role of atomic orbitals in the formation of energy bands, thus affecting the electronic and optical behavior of materials. To investigate the electronic behavior of the investigated compounds, the total density of states (TDOS) was examined, as seen in Figure 6a,d. It offers a broad picture of the available energy states and their distribution along the electronic spectrum. The combined band‐structure analysis and DOS results indicate that both Na_2_TlInF_6_ and K_2_TlInF_6_ exhibit clear semiconducting behavior, with distinct valence and conduction bands separated by an energy gap. The contribution of an individual atomic orbital is known as the partial density of states (PDOS). In Na_2_TlInF_6_ (Figure 6b,c), the *p*‐orbitals of F atoms have maximum contribution to the deep valence band and little contribution of F‐*p* and Tl‐*p* states near the fermi level, with minimum contribution Tl‐*s* states. Although the conduction band is mostly occupied with Tl‐*s* and F‐*p* states, it presented a small peak at 3 and 4.8 eV. Equally, the valence band of K_2_TlInF_6_ (Figure 6e,f) is also dominated by F‐*p* and Tl‐p orbitals, while the conduction band is governed by Tl‐*s* and F‐*p* states, with a minor contribution from the Tl‐p state. These findings suggest that electronic densities near the Fermi level are mainly due to F‐*p* and Tl‐*p* states, which confirm the indirect nature of the bandgap, highlighting their relevance for optoelectronic applications.

**FIGURE 6:**
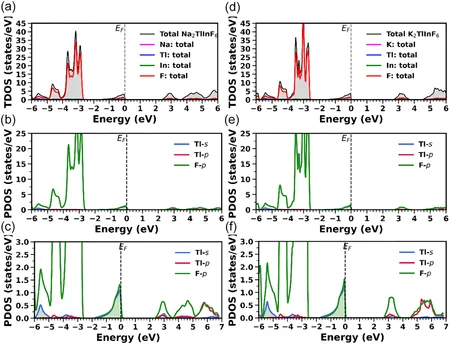
The calculated TDOS (a,d) and PDOS (b,c,e,f) of Na_2_TlInF_6_ and K_2_TlInF_6_ compounds.

The dispersion relation allows us to deduce the effective mass (m*) of photocarriers present at the maxima of the VB and the minima of the CB through the parabolic approximation. The photogenerated carriers of Na_2_TlInF_6_ and K_2_TlInF_6_ undergo thermal relaxation near the extremities of the valence and conduction bands. The effective masses of holes and electrons are derived from the band structure illustrated in Figures 5a–d and S2a,b. Table 4 presents the effective masses of the examined double perovskites. The effective masses slightly decrease with the A‐site cation substitution of Na and K. This indicates that replacing A‐site cations enhances photocarrier mobility. The minimum effective mass of an electron is lower than that of holes, suggesting significant mobility of photogenerated electrons. Although the effective mass of holes has larger values due to the flat valence band dispersion, this suggests a decreased mobility of holes in these materials. Conversely, the mobility of holes associated with electrons is a significant characteristic in the utilization of solar cells. Consequently, it is essential to adjust both the mobility of carriers and the bandgap to attain highly effective solar cells for the compounds under examination. The transport characteristics of semiconductors are governed by the effective masses of charge carriers. Consequently, the effective masses of holes and electrons are determined at the Г–X symmetry points of the valence band maxima and conduction band minima, as seen in Table 4 and Figure S2. In the literature, no findings exist on the examined compounds to compare our results with the masses of charge carriers.

### 3.4 Optical Properties

Evaluating material interactions with electromagnetic radiation and understanding their fundamental electronic structure based on an understanding of their optical characteristics are essential for identifying new materials appropriate for energy‐related applications and high‐performance optoelectronic devices. In this work, the dielectric function, optical conductivity, absorption coefficient, refractive index, and dielectric constant of A_2_TlInF_6_ (A = Na, K) were thoroughly evaluated using the GGA‐PBE potential. The underlying interaction between the material’s electronic states and incident electromagnetic waves is reflected in these highly frequency‐dependent optical responses.

#### 3.4.1 Dielectric Constant

The dielectric function, *ε*(*ω*), provides a basic understanding of a material’s interaction with electromagnetic radiation and supports its optical behavior. It consists of two terms, the real term, $\varepsilon_{1}(\omega )$, the polarizing capacity of a material to an electric field, and the imaginary term, $\varepsilon_{2}(\omega )$, the optical absorption and electronic transitions [78]. This function can be expressed as:

(13) $$\varepsilon \left(\omega \right)=\varepsilon_{1}\left(\omega \right)+i\varepsilon_{2}\left(\omega \right)$$

Whereas,



(14)

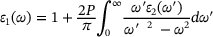

(15) $$\begin{array}{c} \varepsilon_{2}^{\alpha ,\beta }\left(\omega \right)=\frac{4\pi^{2}{е}^{2}}{m^{2}\omega^{2}}\sum_{v,c}\int_{\mathrm{BZ}}^{.}\left\langle vk|p_{\alpha }|ck\right\rangle \left\langle ck|p_{\beta }|vk\right\rangle \\ \delta \left(E_{c}\left(k\right)-E_{v}\left(k\right)-ћ\omega \right)\frac{dk}{{\left(2\pi \right)}^{3}} \end{array}$$

The real and imaginary parts of the dielectric function of A_2_TlInF_6_ (A = Na, K) were evaluated using the TB‐mBJ potential over the photon energy range of 0–13 eV (Figure 7a–d). The $\varepsilon_{1}(\omega )$ spectra show the response of the compounds to external magnetic fields; the first peaks are at about 2.96 eV of Na_2_TlInF_6_ and 3.20 eV of K_2_TlInF_6_. Further with increasing energies, the $\varepsilon_{1}(\omega )$ is dropping at a sharp rate. The other peaks at 3.8–13 eV are observed, and these peaks are associated with the interband transitions. The computed static dielectric constants $\varepsilon_{1}(0)$ are found at 2.45 and 2.29 for Na_2_TlInF_6_ and K_2_TlInF_6_, respectively, depicted in Table 5. The decrease in the value of $\varepsilon_{1}(\omega )$ with Na changed to K is consistent with the Penn model, which indicates that the bandgap has an inverse related with the static dielectric constant. $\varepsilon_{1}(\omega )$ is positive in the entire range of analysis, which means that it shows no change in the dielectric behavior. The imaginary component of the dielectric function, $\varepsilon_{2}(\omega )$, calculated via Equation (15) within the independent‐particle approximation, is governed by direct (vertical) interband transitions. Therefore, the onset of optical absorption does not correspond to the fundamental indirect bandgap, but is governed by the lowest available direct transition in the band structure. For Na_2_TlInF_6_, the lowest direct transition occurs at approximately 2.21 eV, which is slightly lower than the fundamental indirect gap of 2.41 eV. Similarly, for K_2_TlInF_6_, the optical onset is at 2.52 eV of the lowest direct transition observed, while the fundamental indirect gap is 2.68 eV. The sharp increase in $\varepsilon_{2}(\omega )$ beyond these thresholds reflects the high joint density of states for direct transitions at higher energies, reaching to the maximum absorption peaks at 2.32 and 3.45 eV, respectively. These findings indicate that the optical response of these materials is very sensitive to cation substitution, and this directly influences the electronic structure and properties of these materials, rendering them promising materials for optoelectronic applications.

**FIGURE 7:**
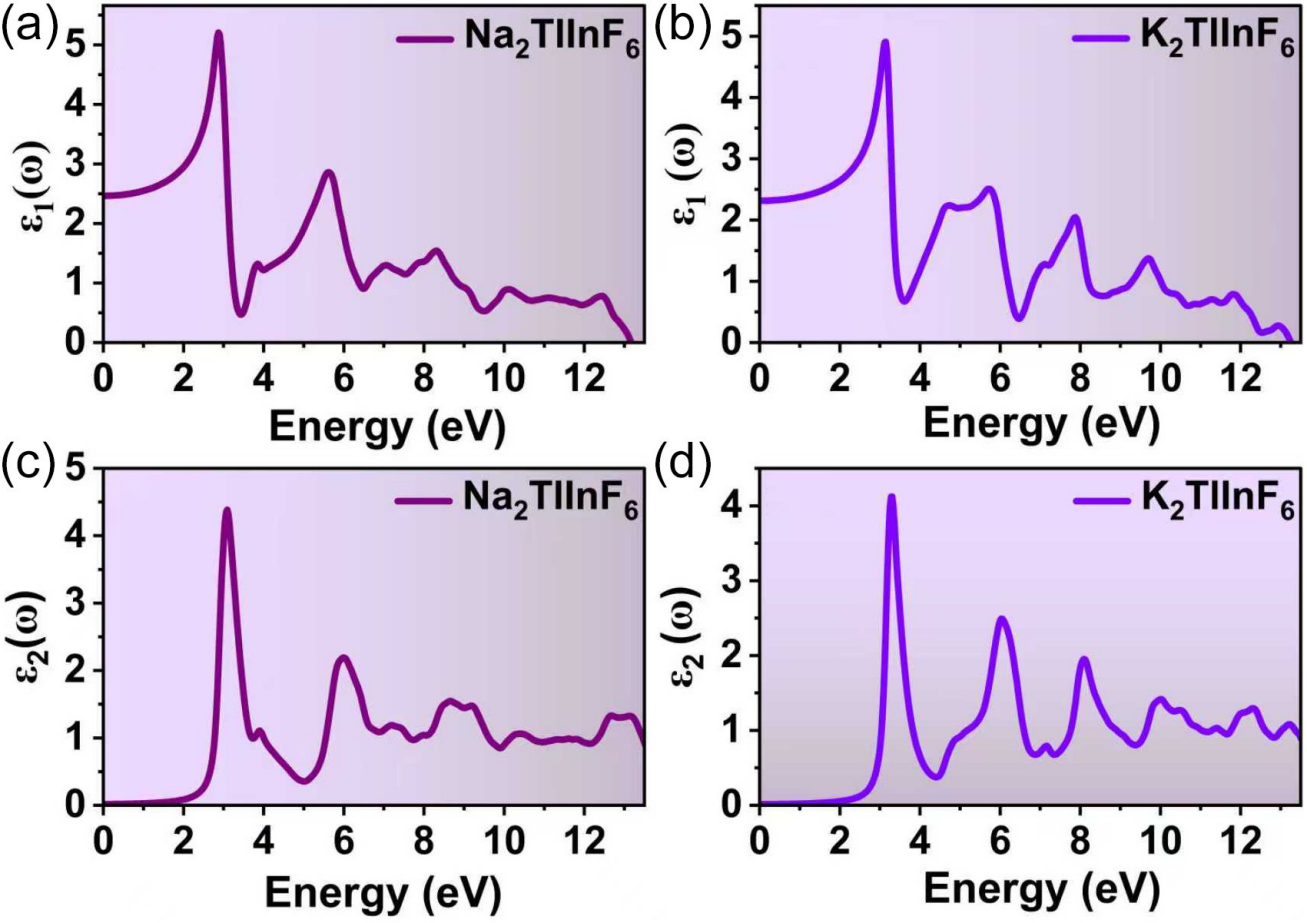
Real and imaginary parts of the dielectric function of Na_2_TlInF_6_ (a,c) and K_2_TlInF_6_ (b,d) perovskites.

**TABLE 5:** Calculated static dielectric function, static refractive index, maximum reflectivity, and absorption coefficient of A_2_TlInF_6_(A = Na, K).

Compounds	*ε*_1_(0)	*n*(0)	*R*_peak_(*ω*)	*I* (*ω*)	References
Na_2_TlInF_6_	2.45	1.55	0.22	120	This work
K_2_TlInF_6_	2.29	1.49	0.20	110	This work
Na_2_InBiF_6_	3.30	1.65	0.17	105.15	[58]
K_2_InBiF_6_	2.83	1.81	0.2	193.68	[58]

#### 3.4.2 Refractive Index

The refractive index is an optical parameter of photoelectric and optoelectronic technologies, and it is the determining factor of the interaction of light with a substance. It measures the rate at which light is slowed down inside the medium and indicates how the material responds to electromagnetic radiation, in terms of frequency dependence and absorption [79]. It is possible to obtain the refractive index, n(ω), by taking the real and imaginary metrics of the dielectric function of ε(ω) respectively, in the following relation:

(16) $$n\left(\omega \right)={\left[\frac{\sqrt{\varepsilon_{1}{\left(\omega \right)}^{2}+\varepsilon_{2}{\left(\omega \right)}^{2}}}{2}+\frac{\varepsilon_{1}{\left(\omega \right)}^{.}}{2}\right]}^{\frac{1}{2}}$$

As shown in Figure 8a,b, the static refractive indices n(ω) of Na_2_TlInF_6_ and K_2_TlInF_6_ are obtained as 1.55 and 1.49, respectively, given in Table 4. The optical spectra show that n(ω) is decreasing with a shift toward Na‐ to K‐based compounds. In addition, the refractive index increases at the zero‐frequency limit, reaching the maximum values of 2.33 at 3.01 eV in Na_2_TlInF_6_ and 2.31 at 3.20 eV in K_2_TlInF_6_. Replacement of Na with K also changes the electronic polarizability, thereby affecting the total optical behavior. Na_2_TlInF_6_ has always shown a higher value of n(ω), so it can be assumed that Na_2_TlInF_6_ exhibits higher polarization and photon–material interaction properties than those of K_2_TlInF_6_.

**FIGURE 8:**
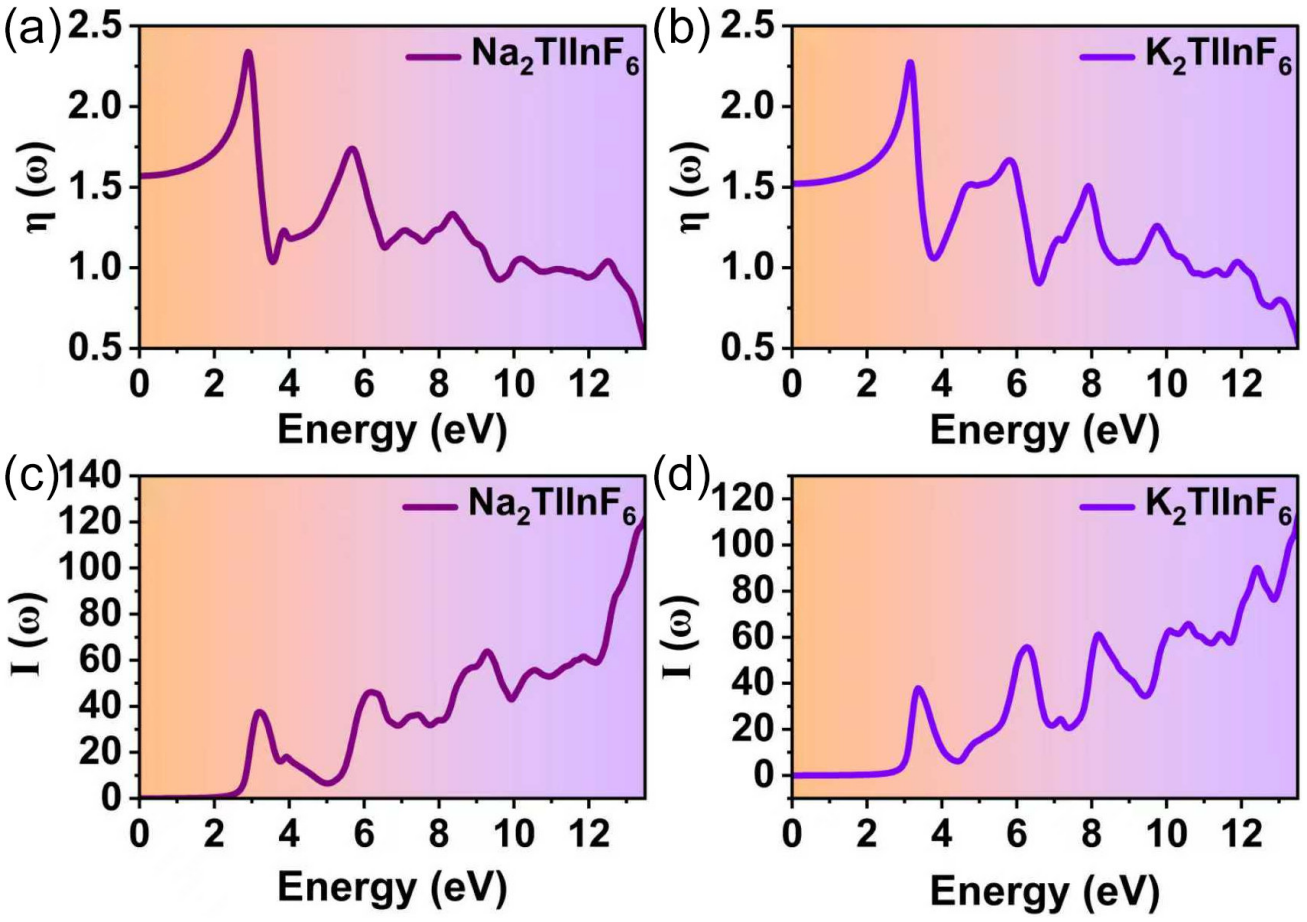
Refractive index (a,b) and absorption coefficient (c,d) of A_2_TlInF_6_ (A = Na, K).

#### 3.4.3 Absorption Coefficient

The absorption coefficient, *I*(*ω*), which measures the amount of light absorbed per unit thickness of a material and provides information about photon‐electron interactions and the material’s energy transition processes, is one of the most significant optical characteristics [79]. One can obtain it based on the dielectric function using the following expression:

(17) I(ω)=2(ω)c[ε1(ω)2+ε2(ω)2−ε1(ω)]12

The observed absorption is due to electronic transitions, both intraband and interband. The obtained A_2_TlInF_6_ (A = Na, K) absorption spectra in Figure 8c,d give a sharp peak at interband electronic transitions at several high‐symmetry points in the Brillouin zone. The computational analysis shows that maximum absorption is observed in the ultraviolet (UV) and visible regions, indicating that the materials could find application in optical devices. The absorption edge is defined as the photon‐energy threshold at which absorption increases significantly. Na_2_TlInF_6_ and K_2_TlInF_6_ exhibit the highest absorption coefficient at the energies of 12.91 and 12.45 eV, respectively, listed in Table 5. Moreover, the total absorption spectrum covers the 2.3–13.0 eV range. K_2_TlInF_6_ exhibits an extended bandgap and strong absorption in the UV region, which should be distinguished from the absorption edges established by the bandgaps. The calculated optical features of these compounds suggest promising optoelectronic uses; their suitability for traditional solar cells or UV‐sensitive devices needs additional experimental and device‐level investigation.

#### 3.4.4 Optical Conductivity

Optical conductivity abbreviated σ(ω) is one of the basic optical properties that characterize how a material carries electric charge as electromagnetic radiation passes through it. It characterizes the material’s behavior to light excitation by measuring the dynamic interaction between incident photons and charge carriers. Optical conductivity is represented as follows:

(18) $$\sigma_{1}\left(\omega \right)=\frac{\omega }{4\pi }\epsilon_{2}\left(\omega \right)$$

The optical conductivity is directly related to imaginary part of dielectric function $\varepsilon_{2}(\omega )$, along with the angular frequency “ω.” A material that allows light to pass through it with little absorption or scattering is said to be transparent; this critical phenomenon is closely related to reflectance. When light shines on the substance, the electrons inside the material can take up the energy of photons and become excited into new electronic states, affecting the optical conductivity of the entire substance. Figure 9a,b shows that the optical conductivity spectra of Na_2_TlInF_6_ and K_2_TlInF_6_ have different peak intensities, indicating distinct optoelectronic responses. Na_2_TlInF_6_ has its maximum conductivity of about 2266.81 Ω cm^−1^ at an energy of 12.61 eV, whereas K_2_TlInF_6_ has a slightly lower maximum of 2140.73 Ω cm^−1^ at 12.38 eV, as shown in Figure 9a,b and Table 5. These results suggest that Na_2_TlInF_6_ interacts more strongly with the electromagnetic field, a factor that enables it to transport charge. In addition, the optical spectra indicate that replacing Na with K shifts the conductivity peak to higher energies, suggesting that the compositional effect is on the optical response of the material’s electronic structure.

**FIGURE 9:**
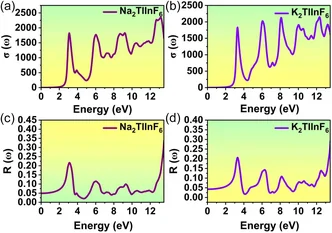
The calculated (a,b) optical conductivity and (c,d) reflectivity of A_2_TlInF_6_ (A = Na, K).

#### 3.4.5 Reflectivity

Reflectivity, R(ω) gives us an understanding of a material’s surface interactions and is a vital factor in optical surface analysis. It is specifically applicable in the study of the electronic structure, bonding structure, and surface morphology of crystalline compounds. The reflectivity may be mathematically defined as:

(19) $$R(\omega )=\frac{n+ik-1}{n+ik+1}$$

where *n* and *k* are the refractive index and the extinction coefficient, respectively. Figure 9c,d demonstrates that Na_2_TlInF_6_ and K_2_TlInF_6_ have relatively sensitive characteristics of reflectance. The materials reflect partial light and have significant transmission and absorption. As listed in Table 5, Na_2_TlInF_6_ and K_2_TlInF_6_ have the highest reflectivity values of about 22% and 20%, respectively. The increased reflectivity of Na_2_TlInF_6_ may also be explained by a greater refractive index and greater interaction between incident photons and electrons on the surface. These properties highlight how the compound’s optical response and surface reflectance are affected by alkali substitution.

In summary, the computed optical characteristics of A_2_TlInF_6_ (A = Na, K) demonstrate their distinct response to incoming electromagnetic radiation, characterized by variations in their dielectric properties, absorption spectra, and optical conductivity. It is suggested that investigated compounds require further experimental and theoretical exploration for prospective uses in wide‐bandgap ultraviolet optoelectronics and tandem solar energy conversion. Nonetheless, the current optical computations are based on an independent‐particle model and do not explicitly incorporate electron–hole interactions (excitonic effects), carrier lifetimes, or defect‐related states, which substantially influence the absorption spectra close to the band edges. Additional research employing techniques like the Bethe–Salpeter equation (BSE) is required to evaluate these actual impacts on the A‍‐‍site substitution.

## 4 Thermodynamic Properties

The thermodynamic characteristics of A_2_TlInF_6_ (A = Na, K) were studied systematically to assess their potential for high‐temperature and mechanical applications. The quasi‐harmonic Debye model was used in the GIBBS package at temperatures up to 1200 K, enabling the evaluation of temperature‐dependent properties based on energy‐volume relations derived with DFT. The Gibbs free energy of both compounds, which is shown in Figure 10a,b, follows a consistent decreasing pattern as the temperature increases, which is characteristic of a thermodynamic system in which, as the systems thermal energy increases, its potential to do work decreases. K_2_TlInF_6_ has always reported higher Gibbs free energy values than Na_2_TlInF_6_ over the temperature range of 0–1200 K.

**FIGURE 10:**
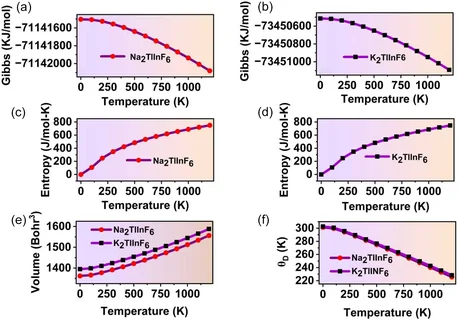
(a,b) Gibbs free energy, (c,d) entropy, and (e,f) volume variations and Debye temperature versus temperature for A_2_TlInF_6_ (A = Na, K).

The temperature dependence of the entropy of the compounds, which are plotted in Figure 10c,d, is expected to be on the same principle, where disorder in a system increases with an increase in thermal energy. Their rate of growth also follows the same pattern, where K_2_TlInF_6_ has a little higher entropy than Na_2_TlInF_6_, which can be explained by the fact that the unit‐cell volume of K_2_TlInF_6_ is higher. The volumetric behavior of the two compounds as a function of temperature, as shown in Figure 10e, indicates that both compounds expand gradually with increasing temperature. This thermal expansion is due to increased atomic vibrations and greater mobility of the constituent particles at high temperatures. Also, the proportion of K_2_TlInF_6_ is higher than that of Na_2_TlInF_6_ under the same thermal conditions, and the volume growth shows an almost linear relationship with the temperature. The Debye temperature, an important parameter for determining the lattice stiffness and thermal conductivity, was also studied as a function of temperature (Figure 10f). Both Na_2_TlInF_6_ and K_2_TlInF_6_ exhibit negative temperature dependence of the Debye temperature, indicating increased atomic vibrations that reduce the lattice rigidity. Notably, K_2_TlInF_6_ presents an elevated Debye temperature over the examined temperature span, suggesting variations in its intrinsic lattice vibrational characteristics relative to Na_2_TlInF_6_. Nonetheless, this difference by itself does not inherently suggest enhanced interatomic bonding, as the elastic characteristics of the two substances exhibit varied patterns. This trend is consistent with earlier observations of similar double‐perovskite systems [80].

## 5 Thermoelectric Properties

The Seebeck coefficient (*S*), electrical conductivity (*σ*), thermal conductivity (*κ*), figure of merit (ZT), and power factor (PF) of A_2_TlInF_6_ (A = Na, K) were obtained based on the semi‐classical Boltzmann transport theory programmed in the BoltzTraP package following the constant relaxation time (*τ* = 10^−14^ s) approximation. The computed thermoelectric properties in the range of 50–800 K are presented in Figures 11 and 12. Bandgap engineering has been suggested as an effective approach to improve thermoelectric efficiency, as the electronic band structure strongly influences thermoelectric performance [81]. The positive Seebeck coefficients calculated for both compounds confirm dominant hole transport, indicating a *p*‐type semiconducting behavior.

**FIGURE 11:**
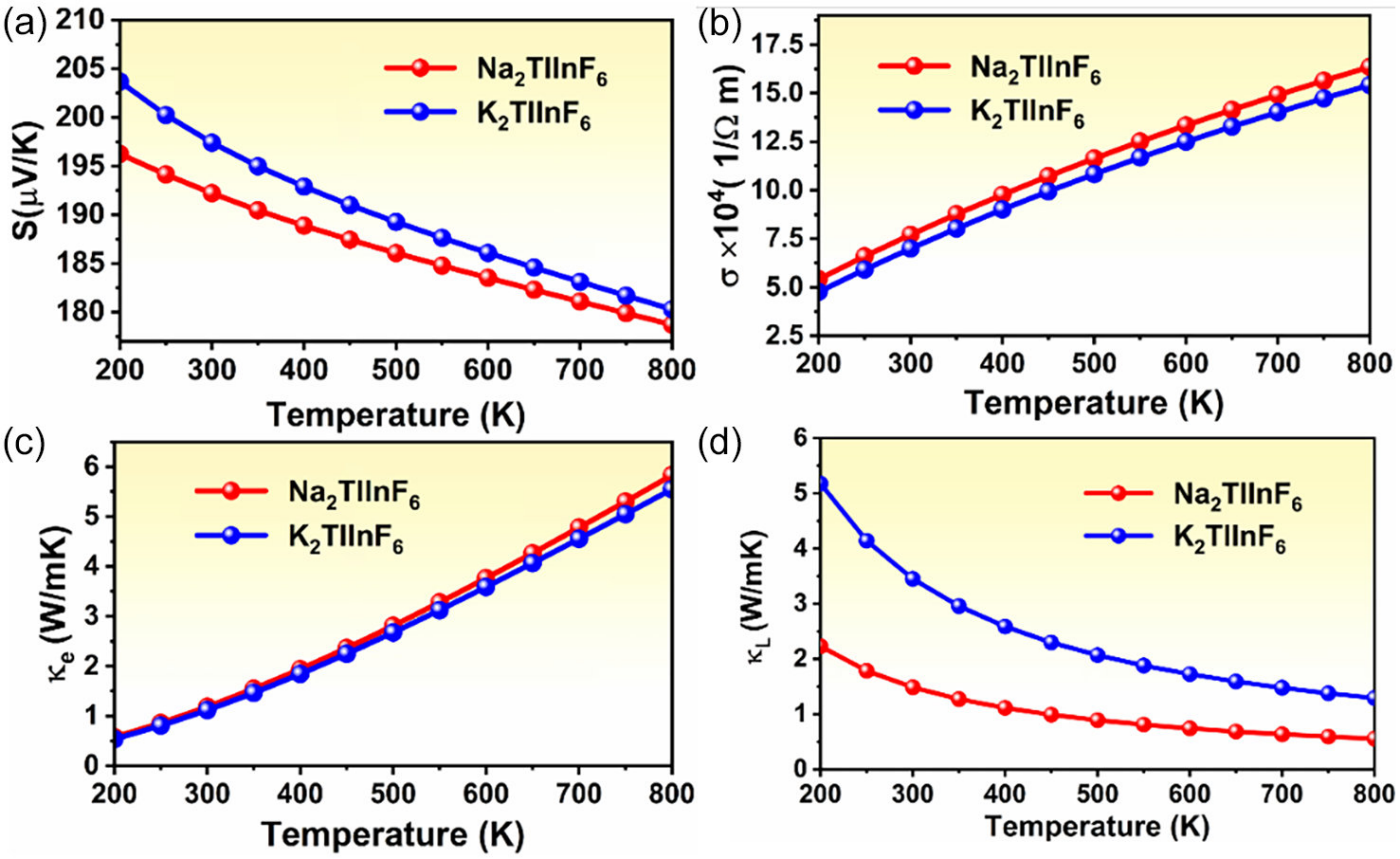
The plots of Seebeck coefficient (a), electrical conductivity (b), and electronic and lattice thermal conductivity (c,d), for A_2_TlInF_6_ (A = Na, K).

**FIGURE 12:**
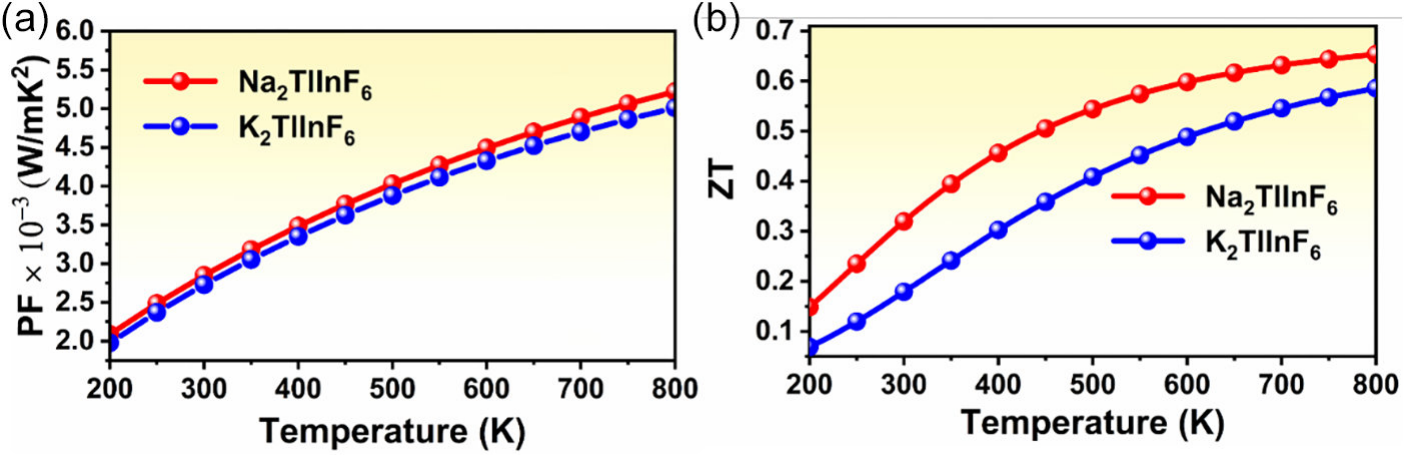
Temperature dependence of (a) figure of merit and (b) power factor for A_2_TlInF_6_ (A = Na, K).

One important factor for evaluating the effectiveness of thermoelectric activities with measuring the potential difference induced by a temperature differential is called the Seebeck coefficient [82, 83]. The thermoelectric efficiency is directly related to the product of Seebeck coefficient and electrical conductivity, resulting in the power factor, PF = *σS* ^2^ [83]. Both Na_2_TlInF_6_ and K_2_TlInF_6_ have Seebeck coefficients of 192.22 and 197.41 μV/K at ambient temperature, respectively. The Seebeck coefficient decreases with increasing temperature up to 800 K, as shown in Figure 11a. At elevated temperature, more charge carriers are thermally excited, resulting in more carriers being produced, which reduce the energy difference between low‐ and high‐energy carriers, and the value of *S* decreases as the temperature rises. Additionally, the studied materials present a *p*‐type semiconducting nature as indicated by the positive value of Seebeck coefficient, which verifies that holes are the predominant charge carriers. Na_2_TlInF_6_ exhibits a higher Seebeck coefficient than K_2_TlInF_6_, which may be attributed to stronger Coulomb interactions and variations in bond strength within the crystal structure. As seen in Figure 11b, the plot presents electrical conductivity per unit of relaxation time (*τ*) versus temperature. The electrical conductivity is a measure of the movement of charge carriers in the conduction band and is strongly affected by their concentration and mobility [84]. At ambient temperature, the “*σ*” metrics are 7.71 × 10^4^ Ω m^−1^ for Na_2_TlInF_6_ and 6.99 × 10^4^ Ω m^−1^ for K_2_TlInF_6_. Both compounds show a close linear increase in electrical conductivity as the temperature is raised, with maximum conductivities of 16.3 × 10^4^ and 15.4 × 10^4^ Ω m^−1^ at 800 K. At high temperatures, the rise in electrical conductivity is due to the enhanced thermal excitation of charge carriers, which is typical behavior of semiconducting materials.

The electronic contribution to the thermal conductivity (*κ*_L_) was computed with the BoltzTraP code, while lattice thermal conductivity is calculated with the slack equation which can be written as [85]:

(20) κL=MavΘD3V13γ2n23TA

In Equation (20), *M*_av_, *γ*, Θ_D_, and A denote the average atomic mass per unit primitive cell, Grüneisen parameter, Debye temperature, and constant, respectively, whereas *V* and *T* represent the fundamental unit cell volume and thermodynamic temperature included in evaluating lattice thermal conductivity. The parameters are ascertained using the formulae provided below. The lattice contribution was computed via the above equation since phonon transport is the limitation of the BoltzTraP code. The total thermal conductivity is generally given by *κ* = *κ*_e_ + *κ*_L_. In this case, where *κ*_e_ and *κ*_L_ represent the electronic and lattice contributions, respectively [86]. As presented in Figure 11c,d, the electronic thermal conductivity, *κ*_e_, follows the Wiedemann–Franz relation: *L* = *κ*_e_/*σ* *T*. The *κ* variations are much higher at high temperatures for both compounds [82]. For Na_2_TlInF_6_, *κ*_e_ increases from 1.18 W/mK at 300 K to 5.83 × 10 W/mK at 800 K, whereas K_2_TlInF_6_ exhibits values ranging from 1.11 to 5.54 W/Km. At maximum temperature, where thermal conductivity increases, there is also an improvement in carrier diffusion and electronic heat transport. Among the compounds studied, Na_2_TlInF_6_ exhibits a higher thermal conductivity relative to K_2_TlInF_6_.

Based on the findings illustrated in Figure 11d, it is clear that the *κ*_L_ values decrease with rising temperature. This occurrence of *κ*_L_ illustrates that phonon‐mediated thermal conduction diminishes with rising temperature. At 300 K, the computed *κ*_L_ was found to be 1.48 and 3.45 W/mK for Na_2_TIInF_6_ and K_2_TIInF_6_, respectively. It is associated with and illustrates the overall thermal transfer via phonons and charge carriers (*κ*) in Figure 11d, which is temperature‐dependent.

Another important quantity employed to evaluate the thermoelectric performance is the power factor, defined as the product of electrical conductivity and the square of Seebeck coefficient (PF = *S* ^2^ *σ*) [87]. The increase in the power factor with temperature is presented in Figure 12a. The linear variation is observed in the PF for both compounds. At ambient temperature, the metrics of 2.84 × 10^−3^ and 2.74 × 10^−3^ W/mK^2^) are observed for Na_2_TlInF_6_ and K_2_TlInF_6_ and increase linearly with rise in temperature, respectively. The behavior indicates that the material possesses enhanced electrical conductivity, leading to an improved power factor at high temperatures. Overall, the thermoelectric properties of Na_2_TlInF_6_ are better than those of K_2_TlInF_6_. Therefore, it is suggested that Na_2_TlInF_6_ has a greater potential for application in thermoelectric energy conversion.

The thermoelectric efficiency of a material is typically expressed by the dimensionless thermoelectric figure of merit (ZT), which is defined as ZT = (*S* ^2^ *T* *σ*/*κ*) [88]. High electrical conductivity, low thermal conductivity, and a high Seebeck coefficient are required for a high ZT value [83]. Materials with ZT values approaching unity or above are considered promising candidates for thermoelectric applications. The calculated ZT metrics are shown as a function of temperature in Figure 12. At ambient temperature, the ZT values of Na_2_TlInF_6_ and K_2_TlInF_6_ are observed as 0.65 and 0.58, respectively. The ZT values at 800 K are almost the same as at ambient temperature, respectively. The ZT values for both compounds approach unity, suggesting their potential for further investigation in thermoelectric applications. However, K_2_TlInF_6_ exhibits a comparatively higher efficiency over the investigated temperature range. The positive Seebeck coefficients, electrical conductivity, and appreciable power factors further support that A_2_TlInF_6_ (A = Na, K) compounds have potential for thermoelectric energy conversion, whereas further optimization is required to improve its efficiency.

## 6 Conclusion

In this work, a comprehensive theoretical investigation of the structural, mechanical, electronic, optical, thermodynamic, and thermoelectric properties of novel double perovskites A_2_TlInF_6_ (A = Na, K) was carried out. The calculated tolerance factors and negative formation energy confirm the structural and energetic stability of the cubic phase for both compounds. Gibbs free energies, together with AIMD simulations, support their energetic and dynamic stability, indicating that both materials are experimentally synthesizable and thermally robust for device fabrication. The mechanical stability and its properties were confirmed with elastic constants, which show mechanical stability, ductility, and an elastic anisotropic nature. The calculated electronic band structure without SOC demonstrates indirect bandgaps of 2.41 and 2.68 eV, respectively. Whereas SOC is included in GGA‐PBE and TB‐mBJ, the corresponding bandgaps are 2.37/5.05 and 2.64/4.75 eV for Na_2_TlInF_6_ and K_2_TlInF_6_, respectively. Moreover, the optical properties demonstrate strong absorption and polarization responses in the photon energy range of 0–13.5 eV, indicating the possible use of the material for capturing visible and ultraviolet light, making them potential candidates for optoelectronic devices such as solar cells, photodetectors, and UV absorbers. From the thermoelectric analysis, it is noticed that the entire Seebeck curve is present in the positive region, which represents the prevailing hole transport and *p*‐type semiconducting nature with 0.65 and 0.58 ZT values for Na_2_TlInF_6_ and K_2_TlInF_6_, respectively. Moreover, the compound Na_2_TlInF_6_ have relatively moderate electrical conductivity, high power factor, and relatively low electronic and lattice thermal conductivity, particularly at high temperature, highlighting viability for thermoelectric applications. The decrease in Gibbs free energy and the rise in entropy with temperature signify advantageous thermodynamic properties, but volume expansion and a declining Debye temperature denote intensified thermal oscillations. The findings suggest that the fluorine‐based double perovskites A_2_TlInF_6_ (A = Na, K) are viable prospects for upcoming thermoelectric and optoelectronic uses, especially in wide‐bandgap ultraviolet optoelectronic devices, tandem solar energy conversion, and waste‐heat reclamation via thermoelectric generators.

## Author Contributions

**Wafa S. Aljuaid:** writing original manuscript, project administration. **Imran Shakir:** manuscript writing, final review. **Shah Zeb Ullah:** assist in investigation. **Shams u Zaman:** data curation. **Johar Zeb:** provide resources. **Syed Hatim Shah:** supervision. **Muhammad Arif:** data analysis. **Dereje Gelanu Dadi:** conceptualization, writing, editing.

## Conflicts of Interest

The authors declare no conflicts of interest.

## Supporting information

Supplementary Material

## Data Availability

The datasets generated and/or analyzed during the current study are available from the corresponding author upon reasonable request.

## References

[1] X. Zheng , C. Liu , Y. Yan , and Q. Wang , “A Review of Thermoelectrics Research–Recent Developments and Potentials for Sustainable and Renewable Energy Applications,” Renewable and Sustainable Energy Reviews 32 (2014): 486–503, 10.1016/j.rser.2013.12.053.

[2] J. Munir , A. S. Jbara , Q. Ain , et al., “An Insight Into the Electronic, Optical and Transport Properties of Promising Zintl‐Phase BaMg_2_P_2_ ,” Physica B: Condensed Matter 618 (2021): 413181, 10.1016/j.physb.2021.413181.

[3] O. K. Varghese and F. L. Souza , Conversion of Water and CO_2_ to Fuels Using Solar Energy: Science, Technology and Materials, (John Wiley & Sons, 2024).

[4] M.‐N. Liu , J.‐H. Liu , L.‐Y. Wang , et al., “Strategies for Improving Contact‐Electro‐Catalytic Efficiency: A Review,” Nanomaterials 15 (2025): 386. 10.3390/nano15050386 PMC11901548 40072189

[5] H. Li , A. Berbille , X. Zhao , Z. Wang , W. Tang , and Z. L. Wang , “A Contact‐Electro‐Catalytic Cathode Recycling Method for Spent Lithium‐Ion Batteries,” Nature Energy 8 (2023): 1137–1144, 10.1038/s41560-023-01348-y.

[6] T. Hammer , T. Kappes , and M. Baldauf , “Plasma Catalytic Hybrid Processes: Gas Discharge Initiation and Plasma Activation of Catalytic Processes,” Catalysis Today 89 (2004): 5–14, 10.1016/j.cattod.2003.11.001.

[7] Z. Lin , W. Shen , J.‐C. Roux , and H. Xi , “Photo‐Catalytic Degradation of Mixed Gaseous HCHO and C_6_H_6_ in Paper Mills: Experimental and Theoretical Study on the Adsorption Behavior Simulation and Catalytic Reaction Mechanism,” Journal of Hazardous Materials 388 (2020): 121779, 10.1016/j.jhazmat.2019.121779. 31848097

[8] M. Atarod , J. Safari , and H. Tebyanian , “Ultrasound Irradiation and Green Synthesized CuO‐NiO‐ZnO Mixed Metal Oxide: An Efficient Sono/Nano‐Catalytic System Toward a Regioselective Synthesis of 1‐Aryl‐5‐Amino‐1 H‐Tetrazoles,” Synthetic Communications 50 (2020): 1993–2006, 10.1080/00397911.2020.1761396.

[9] S. Bose , B. K. Tripathy , A. Debnath , and M. Kumar , “Boosted Sono‐Oxidative Catalytic Degradation of Brilliant Green Dye by Magnetic MgFe_2_O_4_ Catalyst: Degradation Mechanism, Assessment of Bio‐Toxicity and Cost Analysis,” Ultrasonics Sonochemistry 75 (2021): 105592, 10.1016/j.ultsonch.2021.105592. PMC8167203 34049154

[10] J. Munir , M. Jamil , A. S. Jbara , et al., “Spin‐Polarized Electromagnetic and Optical Response of Full‐Heusler Co_2_VZ (Z = Al, Be) Alloys for Spintronic Application,” European Physical Journal Plus 136 (2021): 1009, 10.1140/epjp/s13360-021-01968-x.

[11] S. H. Shah , M. Arif , M. T. Qureshi , et al., Investigation of Structure, Optoelectronic, and Thermodynamic Properties of Gallium‐Based Perovskites GaMF_3_ (M = Ca, and Cd) for Solar Cell Applications, Optical and Quantum Electronics 56 (2024): 1461.

[12] J. Munir , M. Mustafa , H. Naeem , M. Yousaf , E. El‐Shamy , and Q. Ain , “Electronic Structure, Optical and Thermal Response of Lead‐Free RbAuBr_3_ and RbAuBr_4_ Perovskites for Renewable Energy Applications,” ECS Journal of Solid State Science and Technology 11 (2022): 123003, 10.1149/2162-8777/aca795.

[13] I. Haddouch , M. Karouchi , M. Benhamou , and I. Mouallif , “First‐Principles Insights Into Cs_2_LiBH_6_ and K_2_LiBH_6_ Double Perovskites for High‐Performance Hydrogen Storage and Optoelectronic Applications,” Vacuum 253 (2026): 115479.

[14] H. Errahoui , M. Karouchi , A. Ejjabli , et al., “DFT‐Based Investigation of Ca_2_H_3_Br and Ba_2_H_3_I: Electronic, Optical, and Hydrogen Storage Properties for Energy Applications,” Physica Scripta 100 (2025): 125410, 10.1088/1402-4896/ae2992.

[15] A. Hossain , P. Bandyopadhyay , A. Karmakar , et al., “The Hybrid Halide Perovskite: Synthesis Strategies, Fabrications, and Modern Applications,” Ceramics International 48 (2022): 7325–7343, 10.1016/j.ceramint.2021.11.313.

[16] Y. Zhou and W. Chen , “Hybrid Organic–Inorganic Halide Perovskites,” Journal of Applied Physics 128 (2020): 200401, 10.1063/5.0034825.

[17] S. J. Yang , M. Kim , H. Ko , et al., “Visualization and Investigation of Charge Transport in Mixed‐Halide Perovskite via Lateral‐Structured Photovoltaic Devices,” Advanced Functional Materials 28 (2018): 1804067, 10.1002/adfm.v28.41.

[18] E. H. Sepúlveda‐Oviedo , “A Review of Operational Factors Affecting Photovoltaic System Performance,” Energy Conversion and Management: X 26 (2025): 100942, 10.1016/j.ecmx.2025.100942.

[19] G. Li , K. Chen , Y. Cui , et al., “Stability of Perovskite Light Sources: Status and Challenges,” Advanced Optical Materials 8 (2020): 1902012, 10.1002/adom.v8.6.

[20] A. Senocrate , G. Y. Kim , M. Grätzel , and J. Maier , “Thermochemical Stability of Hybrid Halide Perovskites,” ACS Energy Letters 4 (2019): 2859–2870, 10.1021/acsenergylett.9b01605.

[21] S. Ali , X. Wang , S. U. Zaman , et al., “Unlocking Cs_2_GeTmI_6_ Double Perovskite Through DFT‐Simulation: How Strain Shapes Its Electronic and Optical Marvels,” Journal of Materials Science 60 (2025): 12072–12089, 10.1007/s10853-025-11134-8.

[22] S. Ghosh , H. Shankar , and P. Kar , “Recent Developments of Lead‐Free Halide Double Perovskites: A New Superstar in the Optoelectronic Field,” Materials Advances 3 (2022): 3742–3765, 10.1039/D2MA00071G.

[23] A. Laassouli , M. Karouchi , A. Ejjabli , et al., “Structural, Mechanical, Electronic, and Optoelectronic Properties of Wide‐Band‐Gap Hydride Perovskite BaNaH_3_Pt From Hybrid Density Functional Theory,” Next Materials 11 (2026): 101803, 10.1016/j.nxmate.2026.101803.

[24] M. R. Filip , X. Liu , A. Miglio , G. Hautier , and F. Giustino , “Phase Diagrams and Stability of Lead‐Free Halide Double Perovskites Cs_2_BB′ X_6_: B = Sb and, Br, and I,” Journal of Physical Chemistry C 122 (2018): 158–170, 10.1021/acs.jpcc.7b10370.

[25] Z. Gao , G. Mao , S. Chen , et al., “High Throughput Screening of Promising Lead‐Free Inorganic Halide Double Perovskites via First‐Principles Calculations,” Physical Chemistry Chemical Physics 24 (2022): 3460–3469, 10.1039/D1CP04976C. 35076034

[26] X.‐G. Zhao , D. Yang , J.‐C. Ren , Y. Sun , Z. Xiao , and L. Zhang , “Rational Design of Halide Double Perovskites for Optoelectronic Applications,” Joule 2 (2018): 1662–1673, 10.1016/j.joule.2018.06.017.

[27] T. Zhang , Z. Cai , and S. Chen , “Chemical Trends in the Thermodynamic Stability and Band Gaps of 980 Halide Double Perovskites: A High‐Throughput First‐Principles Study,” ACS Applied Materials & Interfaces 12 (2020): 20680–20690, 10.1021/acsami.0c03622. 32281362

[28] H. Absike , N. Baaalla , L. Attou , H. Labrim , B. Hartiti , and H. Ez‐zahraouy , “Theoretical Investigations of Structural, Electronic, Optical and Thermoelectric Properties of Oxide Halide Perovskite ACoO_3_ (A = Nd, Pr or La),” Solid State Communications 345 (2022): 114684, 10.1016/j.ssc.2022.114684.

[29] I. Hamideddine , N. Tahiri , O. E. Bounagui , and H. Ez‐Zahraouy , “Ab Initio Study of Structural and Optical Properties of the Halide Perovskite KBX3 Compound,” Journal of the Korean Ceramic Society 59 (2022): 350–358, 10.1007/s43207-021-00178-6.

[30] I. Hamideddine , H. Zitouni , N. Tahiri , O. El Bounagui , and H. Ez‐Zahraouy , “A DFT Study of the Electronic Structure, Optical, and Thermoelectric Properties of Halide Perovskite KGeI_3_‐* _x_ *Br* _x_ * Materials: Photovoltaic Applications,” Applied Physics A 127 (2021): 443, 10.1007/s00339-021-04600-y.

[31] K. Li , S. Li , W. Zhang , et al., “Highly‐Efficient and Stable Photocatalytic Activity of Lead‐Free Cs_2_AgInCl_6_ Double Perovskite for Organic Pollutant Degradation,” Journal of Colloid and Interface Science 596 (2021): 376–383, 10.1016/j.jcis.2021.03.144. 33848744

[32] Y. Wang , H. Huang , Z. Zhang , et al., “Lead‐Free Perovskite Cs_2_AgBiBr_6_@ g‐C_3_N_4_ Z‐Scheme System for Improving CH_4_ Production in Photocatalytic CO_2_ Reduction,” Applied Catalysis B: Environmental 282 (2021): 119570, 10.1016/j.apcatb.2020.119570.

[33] A. H. Slavney , T. Hu , A. M. Lindenberg , and H. I. Karunadasa , “A Bismuth‐Halide Double Perovskite With Long Carrier Recombination Lifetime for Photovoltaic Applications,” Journal of the American Chemical Society 138 (2016): 2138–2141, 10.1021/jacs.5b13294. 26853379

[34] T. Wang , D. Yue , X. Li , and Y. Zhao , “Lead‐Free Double Perovskite Cs_2_AgBiBr_6_/RGO Composite for Efficient Visible Light Photocatalytic H_2_ Evolution,” Applied Catalysis B: Environment and Energy 268 (2020): 118399, 10.1016/j.apcatb.2019.118399.

[35] J. Luo , S. Li , H. Wu , et al., “Cs_2_AgInCl_6_ Double Perovskite Single Crystals: Parity Forbidden Transitions and Their Application for Sensitive and Fast UV Photodetectors,” ACS Photonics 5 (2018): 398–405, 10.1021/acsphotonics.7b00837.

[36] K. Lv , S. Qi , G. Liu , Y. Lou , J. Chen , and Y. Zhao , “Lead‐Free Silver‐Antimony Halide Double Perovskite Quantum Dots With Superior Blue Photoluminescence,” Chemical Communications 55 (2019): 14741–14744, 10.1039/C9CC07397C. 31754680

[37] Y. Cai , W. Xie , Y. T. Teng , et al., “High‐Throughput Computational Study of Halide Double Perovskite Inorganic Compounds,” Chemistry of Materials 31 (2019): 5392–5401, 10.1021/acs.chemmater.9b00116.

[38] X.‐G. Zhao , J.‐H. Yang , Y. Fu , et al., “Design of Lead‐Free Inorganic Halide Perovskites for Solar Cells via Cation‐Transmutation,” Journal of the American Chemical Society 139 (2017): 2630–2638, 10.1021/jacs.6b09645. 28112933

[39] J. C. Dahl , W. T. Osowiecki , Y. Cai , et al., “Probing the Stability and Band Gaps of Cs_2_AgInCl_6_ and Cs_2_AgSbCl_6_ Lead‐Free Double Perovskite Nanocrystals,” Chemistry of Materials 31 (2019): 3134–3143, 10.1021/acs.chemmater.8b04202.

[40] J. Zhou , X. Rong , M. S. Molokeev , X. Zhang , and Z. Xia , “Exploring the Transposition Effects on the Electronic and Optical Properties of Cs_2_AgSbCl_6_ via a Combined Computational‐Experimental Approach,” Journal of Materials Chemistry A 6 (2018): 2346–2352, 10.1039/C7TA10062K.

[41] W. Deng , Z.‐Y. Deng , J. He , et al., “Synthesis of Cs_2_AgSbCl_6_ and Improved Optoelectronic Properties of Cs_2_AgSbCl_6_/TiO_2_ Heterostructure Driven by the Interface Effect for Lead‐Free Double Perovskites Solar Cells,” Applied Physics Letters 111 (2017): 151602, 10.1063/1.4999192.

[42] D.‐Y. Hu , X.‐H. Zhao , T.‐Y. Tang , et al., “First‐Principles Study on the Structural, Elastic, Electronic and Optical Properties of Lead‐Free Double Perovskites Cs_2_CuBiX_6_ (X = I, Br, Cl),” Materials Today Communications 29 (2021): 102842, 10.1016/j.mtcomm.2021.102842.

[43] J. Dai , L. Ma , M. Ju , J. Huang , and X. C. Zeng , “In‐and Ga‐Based Inorganic Double Perovskites With Direct Bandgaps for Photovoltaic Applications,” Physical Chemistry Chemical Physics 19 (2017): 21691–21695, 10.1039/C7CP03448B. 28770937

[44] S. M. Qaid , H. Murtaza , Q. ul Ain , et al., “A Computational Approach to Correlate the Physical Attributes of Lead‐Free Rb_2_XRhF_6_ (X = Li, Ag) Double Perovskite Halides for Optoelectronics and Renewable Energy Applications,” Physica B: Condensed Matter 671 (2023): 415416, 10.1016/j.physb.2023.415416.

[45] S. M. Qaid , H. Murtaza , Q. Ain , et al., “The Physical Attributes of Rubidium‐Based Rb_2_TlRhF_6_ Double Perovskite Halide: A First‐Principles Investigation,” Physica B: Condensed Matter 685 (2024): 416000, 10.1016/j.physb.2024.416000.

[46] H. Murtaza , Q. Ain , A. S. Jbara , et al., A DFT‐Based Determination of the Physical Attributes of Cs_2_InBiF_6_ Fluoroperovskite by Using Multiple Potentials, Optical and Quantum Electronics 56 (2024): 1440.

[47] G. Murtaza , T. Alshahrani , R. A. Khalil , et al., “Lead Free Double Perovsites Halides X_2_AgTlCl_6_ (X= Rb, Cs) for Solar Cells and Renewable Energy Applications,” Journal of Solid State Chemistry 297 (2021): 121988, 10.1016/j.jssc.2021.121988.

[48] M. Delor , A. H. Slavney , N. R. Wolf , et al., “Carrier Diffusion Lengths Exceeding 1 μm Despite Trap‐Limited Transport in Halide Double Perovskites,” ACS Energy Letters 5 (2020): 1337–1345, 10.1021/acsenergylett.0c00414.

[49] H. Murtaza , Q. Ain , J. Munir , H. M. Ghaithan , A. A. A. Ahmed , and S. M. Qaid , “First‐Principles Analysis on the Optoelectronic, Structural, Elastic and Transport Characteristics of Novel Fluoroperovskites Cs_2_TlAgF_6_ for Green Technology,” ECS Journal of Solid State Science and Technology 13 (2024): 033006, 10.1149/2162-8777/ad3003.

[50] M. Manzoor , M. W. Iqbal , N. A. Noor , H. Ullah , R. Sharma , and S. S. Alarfaji , “Exploring the Structural, Electronic, Optical, and Thermoelectric Properties of Potassium‐Based Double Perovskites K_2_AgXI_6_ (X = Sb, Bi) Compounds: A DFT Study,” Materials Science and Engineering: B 287 (2023): 116122, 10.1016/j.mseb.2022.116122.

[51] I. J. Mbonu , H. Louis , U. G. Chukwu , E. C. Agwamba , S. Ghotekar , and A. S. Adeyinka , “Effects of Metals (X = Be, Mg, Ca) Encapsulation on the Structural, Electronic, Phonon, and Hydrogen Storage Properties of KXCl_3_ Halide Perovskites: Perspective From Density Functional Theory,” International Journal of Hydrogen Energy 50 (2024): 337–351, 10.1016/j.ijhydene.2023.07.099.

[52] M. Zanib , M. W. Iqbal , M. Manzoor , et al., “A DFT Investigation of Mechanical, Optical and Thermoelectric Properties of Double Perovskites K_2_AgAsX_6_ (X= Cl, Br) Halides,” Materials Science and Engineering: B 295 (2023): 116604, 10.1016/j.mseb.2023.116604.

[53] P. Modak and B. Modak , “Energetic, Electronic, and Optical Properties of Intrinsic Charge Carrier‐Trapping Defects in KTaO_3_: Insights From a Hybrid DFT Study,” Journal of Physical Chemistry C 125 (2021): 24067–24078, 10.1021/acs.jpcc.1c06092.

[54] S. Wang , Y. Chen , B. Cui , et al., “The Combined First Principles and Experimental Study of O‐Doped KMnF_3_ as a Cathode for Potassium‐Ion Batteries,” Applied Surface Science 514 (2020): 145954, 10.1016/j.apsusc.2020.145954.

[55] R. Anbarasan , M. Srinivasan , J. K. Sundar , et al., “The Effect of Anion Exchange on the Electronic and Optical Properties of Vacancy Ordered Double Perovskites K_2_PdX_6_ (X = Cl, Br, I) Using First Principle Calculations,” Molecular Simulation 48 (2022): 1527–1532, 10.1080/08927022.2022.2103161.

[56] S. H. Shah , P. Song , T. Huang , et al., “Uniaxial Strain Engineering of Electronic, Elastic and Optical Properties of Halide Double Perovskites K_2_NaTIX_6_ (X = I, Br, and Cl): A DFT Insight,” Journal of Inorganic and Organometallic Polymers and Materials 35 (2025): 3665–3681, 10.1007/s10904-024-03484-8.

[57] H. Murtaza , Q. Ain , J. Munir , et al., “Tuning the Thermoelectric and Optoelectronic Attributes of Lead‐Free Novel Fluoroperovskites Cs_2_BB’F_6_ (B = Rb, in, Na and B’ = Ir, As, Rh): A First‐Principles Investigation,” Journal of Physics and Chemistry of Solids 190 (2024): 111934, 10.1016/j.jpcs.2024.111934.

[58] A. El Mekkaouy , E. H. Akarchaou , S. Chtita , A. Sali , H. Khajmi , and R. Touti , “First‐Principles Analysis of X_2_InBiF_6_ (X = Na, K and Rb) Fluoroperovskites for Optoelectronic and Photovoltaic Applications,” Next Materials 12 (2026): 102317, 10.1016/j.nxmate.2026.102317.

[59] S. Ahmad , M. Arif , S. Ali , et al., “DFT Study on Elastic, Structural, and Optoelectronic Properties of Cs_2_KAsA_6_ (A = Cl, Br, I) Halide Double Perovskites: Potential for Green Energy Applications,” Journal of Inorganic and Organometallic Polymers and Materials 35 (2025): 7734–7745, 10.1007/s10904-025-03781-w.

[60] S. Chelli , S. Touam , N. Mounis , et al., “Ab‐Initio Analysis of the Structural, Electronic, Elastic, and Optical Characteristics of Y* _x_ *B_1_–* _x_ *As Alloys Using the FP‐LAPW Approach,” Physics of the Solid State 67 (2025): 1120–1134, 10.1134/S1063783425602875.

[61] B. K. Sadr , A. Shokri , and B. A. Ravan , “Comparative Study of TB‐mBJ and GGA‐PBE Techniques on the Electronic and Optical Characteristics of Al* _x_ *Ga_1_ _− *x* _N Alloys,” Results in Physics 76 (2025): 108417, 10.1016/j.rinp.2025.108417.

[62] M. Lebeda , J. Drahokoupil , P. Veřtát , et al., “XRDlicious: An Interactive Web‐Based Platform for Online Calculation of Diffraction Patterns and Radial Distribution Functions From Crystal Structures,” Applied Crystallography 58 (2025): 1810, 10.1107/S1600576725005370. PMC12502867 41064442

[63] L. Ji , J. Kou , S. Wang , et al., “Octahedral Cs_2_AgBiBr_6_ Double Perovskite Microcrystals for C─C Coupling Reactions Under Visible‐Light Irradiation,” Inorganic Chemistry 64 (2025): 18401–18410, 10.1021/acs.inorgchem.5c02942. 40891087

[64] M. A. Ali , H. Zarin , S. Salam , et al., “DFT Investigations of Structural, Magnetic, Electronic, and Optical Properties of CsEuCl_3_ ,” Journal of Superconductivity and Novel Magnetism 33 (2020): 1045–1051, 10.1007/s10948-019-05291-6.

[65] S. Maqsood , S. Mumtaz , and Y. Alanazi , “Opto‐Electronic and Thermoelectric Properties of Double Perovskites Na 2 AgGaY 6 (Y= Cl, Br, I) for Energy Conversion Applications: DFT Calculations,” Digest Journal of Nanomaterials & Biostructures 19 (2024): 919.

[66] A. Ayyaz , M. Irfan , M. B. Shakir , et al., “First Principles Study of Optoelectronic, Thermoelectric, and Mechanical Features of Double Perovskites K_2_AgGaZ_6_ (Z = Cl, Br) for Energy Harvesting Perspectives,” Optical and Quantum Electronics, 56 (2024): 1419.

[67] S. Shakeel , P. Song , H. A. Alsalmah , G. Murtaza , and T. Huang , “Investigation of Pressure‐Dependent Electronic and Optical Properties of Double Perovskites Cs_2_AgXY_6_ (X = Bi, In; Y = Cl, Br),” Journal of Inorganic and Organometallic Polymers and Materials 34 (2024): 1040–1054, 10.1007/s10904-023-02888-2.

[68] N. R. Kumar and R. Radhakrishnan , “Electronic, Optical and Mechanical Properties of Lead‐Free Halide Double Perovskites Using First‐Principles Density Functional Theory,” Materials Letters 227 (2018): 289–291, 10.1016/j.matlet.2018.05.082.

[69] Y. Guo , Y. Lou , J. Chen , and Y. Zhao , “Lead‐Free Cs_2_AgSbCl_6_ Double Perovskite Nanocrystals for Effective Visible‐Light Photocatalytic C─C Coupling Reactions,” ChemSusChem 15 (2022): e202102334, 10.1002/cssc.v15.3. 34898013

[70] M. K. Masood , W. Khan , K. Chaoui , et al., “Theoretical Investigation of XSnH_3_ (X: Rb, Cs, and Fr) Perovskite Hydrides for Hydrogen Storage Application,” International Journal of Hydrogen Energy 63 (2024): 1248–1257, 10.1016/j.ijhydene.2024.03.229.

[71] A. Ayyaz , M. A. Ullah , M. Zaman , et al., “Investigation of Hydrogen Storage and Energy Harvesting Potential of Double Perovskite Hydrides A_2_LiCuH_6_ (A = Be/Mg/Ca/Sr): A DFT Approach,” International Journal of Hydrogen Energy 102 (2025): 1329–1339, 10.1016/j.ijhydene.2025.01.117.

[72] A. EL Haji , M. Karouchi , A. Ejjabli , et al., “First‐Principles Investigation of Structural, Mechanical, Electronic, Optical, and Thermodynamic Properties of Perovskite Hydrides XRuH_3_ (X = Mg, Ca, Sr, Ba) for Hydrogen Storage,” International Journal of Hydrogen Energy 223 (2026): 154379, 10.1016/j.ijhydene.2026.154379.

[73] C. J. Bartel , C. Sutton , B. R. Goldsmith , et al., “New Tolerance Factor to Predict the Stability of Perovskite Oxides and Halides,” Science Advances 5 (2019): eaav0693, 10.1126/sciadv.aav0693. PMC6368436 30783625

[74] S. A. Khandy , T. Alshahrani , H. Elsaeedy , and D. C. Gupta , “First‐Principles Calculations to Investigate Structural, Electronic, Phonon, Magnetic and Thermal Properties of Stable Halide Perovskite Semiconductors Cs_2_GeMnI_6_ and Cs_2_GeNiI_6_ ,” Journal of Alloys and Compounds 957 (2023): 170296, 10.1016/j.jallcom.2023.170296.

[75] J. Nematollahi , H. Gharagoozloo , S. Jalali‐Asadabadi , and M. Jamal , “Elastic Constants and Their Variation by Pressure in the Cubic PbTiO_3_ Compound Using IRelast Computational Package Within the Density Functional Theory,” Iranian Journal of Physics Research 19 (2019): 37–48, 10.29252/ijpr.19.1.5.

[76] A. Ayyaz , G. Murtaza , M. Shafiq , M. Q. Shah , N. Sfina , and S. Ali , “Exploring Structural, Thermodynamic, Elastic, Electro‐Optic, and Thermoelectric Characteristics of Double Perovskites Rb_2_XInBr_6_ (X = Na, K) for Photovoltaic Applications: A DFT Approach,” Solar Energy 265 (2023): 112131, 10.1016/j.solener.2023.112131.

[77] R. Ahmad , N. Mehmood , S. Bukhari , W. Nafees , and M. Arif , “Ab Initio Study of Half‐Heusler Compounds MnVZ (Z = P, As, Sb),” Journal of Superconductivity and Novel Magnetism 31 (2018): 2617–2627, 10.1007/s10948-017-4500-z.

[78] X. Diao , Y. Tang , Q. Gu , Y. Shi , and P. Zhu , “The Structural, Stability, Electronic and Optoelectronics Properties of Y_2_AgBiX_6_ (Y = K, Na, Li; X = I, Br, Cl) Halide Double Perovskites: A First‐Principles Study,” Solar Energy 262 (2023): 111914, 10.1016/j.solener.2023.111914.

[79] I. M. Alsalamah , A. Shaari , N. A. Alsaif , S. A. Yamusa , G. Lakshminarayana , and N. Rekik , “Exploring the Structural Properties and the Optoelectronic Features of RbPbX_3_ (X = Cl, F) Perovskite Crystals for Solar Cells Solicitations: Showcasing the DFT Predictions,” Chemical Physics 573 (2023): 111978, 10.1016/j.chemphys.2023.111978.

[80] W. Kuang , H. Wang , X. Li , J. Zhang , Q. Zhou , and Y. Zhao , “Application of the Thermodynamic Extremal Principle to Diffusion‐Controlled Phase Transformations in Fe‐CX Alloys: Modeling and Applications,” Acta Materialia 159 (2018): 16–30, 10.1016/j.actamat.2018.08.008.

[81] Y. Wang , W. Lian , and Y. Liu , “Electronic Structure and Optical Properties in Alkaline‐Earth Metals (A = Mg, Ca, Ba) and Ir Co‐Doped SrTiO_3_: A DFT+ U Investigation,” Optik 228 (2021): 166128, 10.1016/j.ijleo.2020.166128.

[82] D. Moço , J. F. Malta , L. F. Santos , E. B. Lopes , and A. P. Gonçalves , “Thermoelectric Properties of Nickel and Selenium Co‐Doped Tetrahedrite,” Materials 16 (2023): 898, 10.3390/ma16030898. PMC9917858 36769905

[83] M. B. Uddin , M. H. K. Rubel , S. Bouhmaidi , et al., “Thallium‐Based Iodo‐Perovskites TlMI_3_ (M = Ca, Sr) for Optoelectronic and Thermoelectric Applications: A DFT Investigation,” Journal of Physics and Chemistry of Solids 217 (2026): 113796, 10.1016/j.jpcs.2026.113796.

[84] H. Labrim , A. Jabar , L. Laanab , et al., “Optoelectronic and Thermoelectric Properties of the Perovskites: NaSnX_3_ (X = Br or I)—A DFT Study,” Journal of Inorganic and Organometallic Polymers and Materials 33 (2023): 3049–3059, 10.1007/s10904-023-02788-5.

[85] S. H. Shah , T. Huang , S. Shakeel , S. Khan , M. W. Ashraf , and G. Murtaza , “Comprehensive Study of Structural, Elastic, Electronic, Optical, and Thermoelectric Properties of Rb_2_NaTlZ_6_ (Z = Cl, Br, and I) by DFT,” Materials Science in Semiconductor Processing 178 (2024): 108400.

[86] M. Asghar , M. Zanib , M. A. Khan , S. Niaz , N. Noor , and A. Dahshan , “Tuning of the Bandgap of Rb_2_ScAgX_6_ (X = Cl, Br, I) Double Perovskites Through Halide Ion Replacement for Solar Cell Applications,” Materials Science in Semiconductor Processing 148 (2022): 106819.

[87] D. Behera and S. K. Mukherjee , “Optoelectronics and Transport Phenomena in Rb_2_InBiX_6_ (X = Cl, Br) Compounds for Renewable Energy Applications: A DFT Insight,” Chemistry 4 (2022): 1044–1059, 10.3390/chemistry4030071.

[88] S. Berri , “Thermoelectric Properties of A_2_BCl_6_: A First Principles Study,” Journal of Physics and Chemistry of Solids 170 (2022): 110940, 10.1016/j.jpcs.2022.110940.

