# Supplementary material for: Exploring A‐Site Chemistry of A2TlInF6 (A = Na, K) Double Perovskites: Structural, Optoelectronic, Elastic, and Thermoelectric Properties
Source: ChemistryOpen. 2026 Nov 11;15(11):e70342. doi: 10.1002/open.70342 (PMC13755683; doi:10.1002/open.70342)
Supplement: Supplementary file 1 [file OPEN-15-e70342-s001.pdf]

Exploring A-site chemistry of A_2TlInF_6 (A = Na, K) Double Perovskites: Structural, Optoelectronic, Elastic, and Thermoelectric properties

Wafa S Aljuaid[1], Imran Shakir[2], Shah Zeb Ullah [3], Shams u Zaman [4], Johar Zeb[5], Syed Hatim Shah[5], Muhammad Arif [6]*, Dereje Gelanu Dadi [7]*

[1] *Department of Chemistry, Taif University, Taif 26571, Saudi Arabia*

[2] *Department of Physics, Faculty of Science, Islamic University of Madinah, Madinah 42351, Saudi Arabia*

[3]*Department of physics, Government postgraduate College Kohat, Kohat 26000, KP, Pakistan*

[4]*Department of Physics, Kohat University of Science and Technology, Kohat 26000, Pakistan*

[5]*Department of Physics, School of Physics and Astronomy, Yunnan University, Kunming, 650504, China*

[6]*School of Physics and Electronic Information, Yunnan Normal University, Kunming 650500, Yunnan, China*

[7]*Department of Physics, College of Natural and Computational Science, Dambi Bollo University, P.O. Box 260, Dambi Dollo, Ethiopia*

Corresponding author; Syed Hatim Shah (syed_hatim@yahoo.com), Muhammad Arif (arifseher6@gamil.com), Dereje Gelanu Dadi (dereje.gelanu@astu.edu.et)

Supplementary Materials

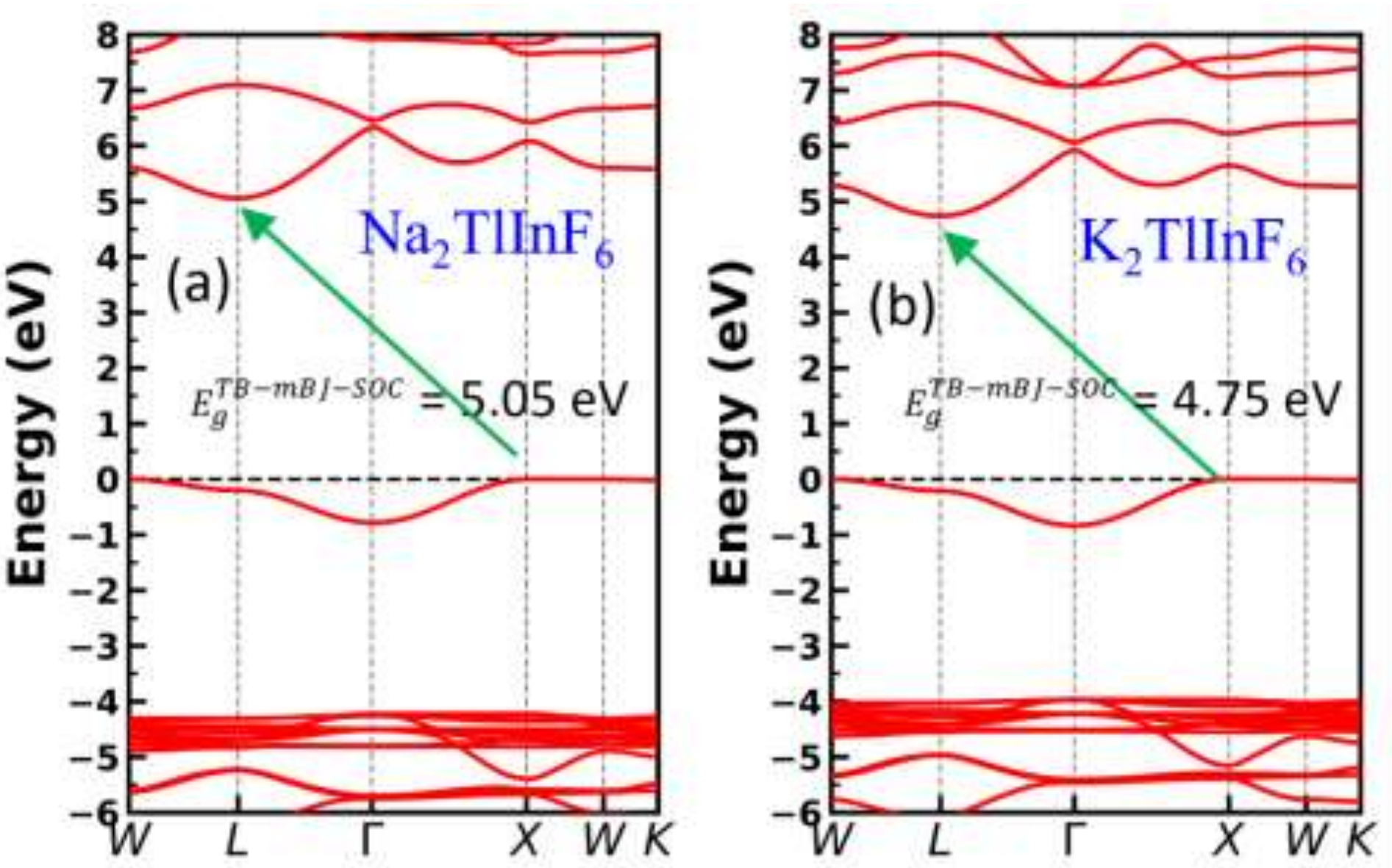

Fig. S1. (a, b) Electronic band structures of Na_2TlInF_6 and K_2TlInF_6 calculated using TB-mBJ-SOC, respectively.

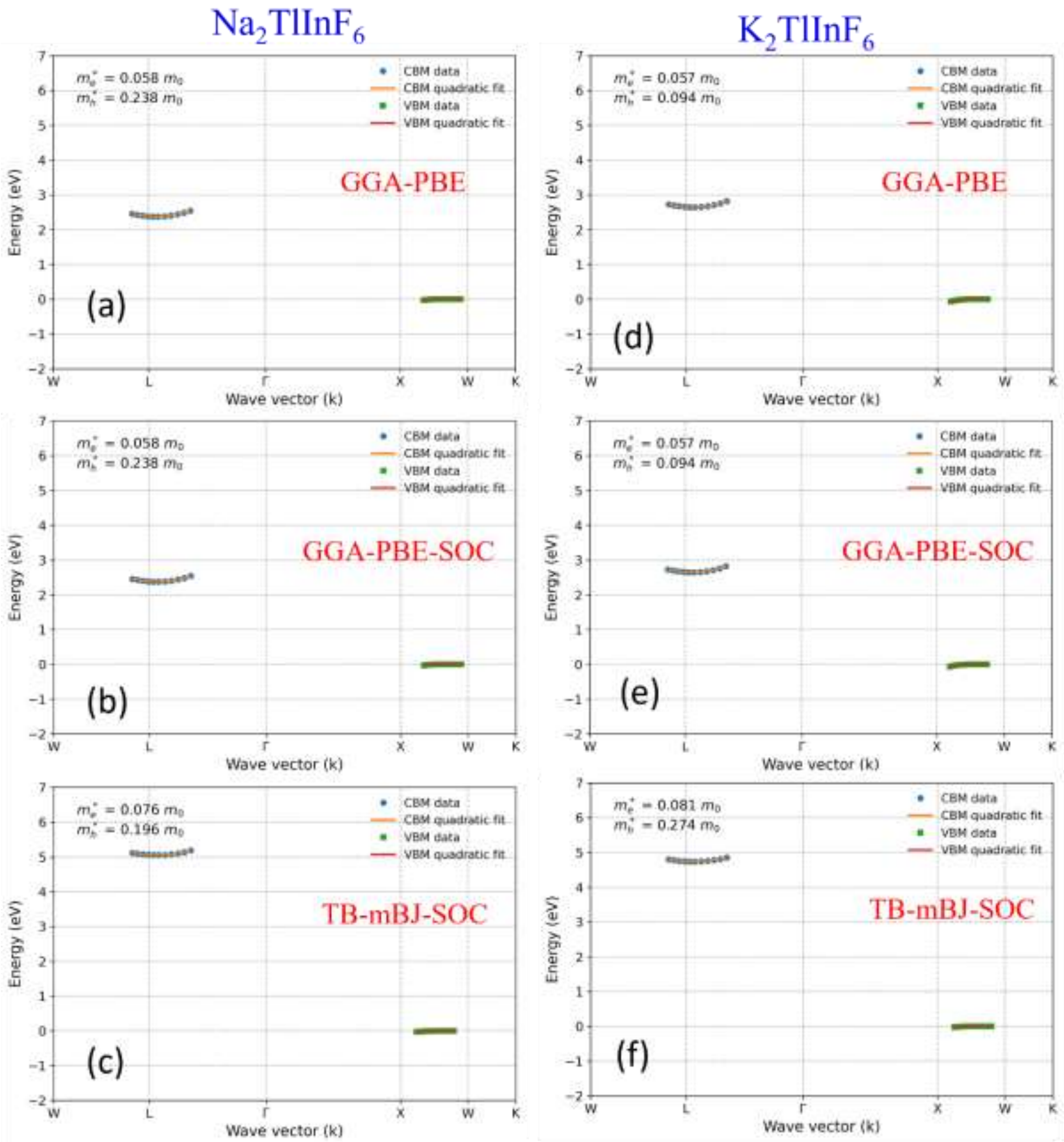

Fig. S2. (a-c) Calculated effective masses of Na_2TlInF_6 and (d-f) K_2TlInF_6 using GGA-PBE, GGA-PBE-SOC, and TB-mBJ-SOC approximations, respectively.
